# Inductive Conformal Prediction for Guaranteed Class-Label Coverage in Object Detection

**DOI:** 10.3390/jimaging12080348

**Published:** 2026-08-02

**Authors:** Mohammed Aliy Mohammed, Esla Timothy Anzaku, Jef Jonkers, Janarthanan Krishnamoorthy, Wesley De Neve, Sofie Van Hoecke

**Affiliations:** 1 IDLab, Department of Electronics and Information Systems, Ghent University, 9052 Ghent, Belgium; eslatimothy.anzaku@ugent.be (E.T.A.); jef.jonkers@ugent.be (J.J.); wesley.deneve@ghent.ac.kr (W.D.N.); sofie.vanhoecke@ugent.be (S.V.H.); 2 School of Biomedical Engineering, Jimma University, Jimma P.O. Box 378, Ethiopia; janarthanan.krishnamoorthy@ju.edu.et; 3 Center for Biosystems and Biotech Data Science, Ghent University Global Campus, Incheon 21985, Republic of Korea; 4 IDLab, imec, 9052 Ghent, Belgium

**Keywords:** conformal prediction, object detection, image classification, uncertainty quantification

## Abstract

Conformal prediction has emerged as a principled framework for uncertainty quantification in computer vision, offering rigorous finite-sample coverage guarantees. However, its application in object detection has remained largely confined to localization, as standard inference codebases typically yield only top-1 class scores, precluding full class-label conformalization. In this work, we bridge this gap by adapting four architecturally diverse detectors—Faster R-CNN, RetinaNet, YOLO11, and RT-DETRv2—to facilitate the extraction of comprehensive per-class score vectors and the estimation of background confidence in the absence of native background modeling. Leveraging these adapted architectures, we implement inductive conformal prediction (ICP) using five distinct nonconformity functions: Top-K, Adaptive Prediction Sets (APS), Hinge, Margin, and Brier score. Our framework is rigorously benchmarked across a curated 20-class subset of MS-COCO and two specialized parasite egg datasets (AI4NTD P1.5v2 and Chula-ParasiteEgg-11). In addition, a Naive cumulative-threshold method is included as a baseline for comparison with APS, given their comparable mathematical formulations. Across target coverage levels of 90%, 95%, and 99%, the conformalized models consistently achieved nominal coverage with only minor finite-sample deviations. Hinge and APS exhibited an optimal balance between statistical coverage and prediction-set efficiency, whereas Margin and Brier scores tended toward larger sets under high data complexity and strict coverage requirements. With empty prediction sets maintained below 0.1%, our findings establish ICP as a robust and adaptable paradigm for trustworthy class-label uncertainty estimation, particularly within safety-critical workflows such as automated parasite diagnostics.

## 1. Introduction

Object detection, a fundamental problem in computer vision, requires the precise localization and identification of objects within an image [1]. This technology has proven indispensable across a broad range of applications, including autonomous driving [2], extreme-environment perception [3,4], security and surveillance [5], healthcare [6], retail and e-commerce [7,8], agriculture [9,10], manufacturing [11], sports [12], environmental monitoring [13,14], and robotics [15]. Despite their widespread adoption, object detection models, like other machine learning techniques, are inherently subject to both aleatoric and epistemic uncertainty. Aleatoric uncertainty, arising from intrinsic variability or noise within the training data, and epistemic uncertainty, stemming from model limitations or the constraints imposed by finite data sampling, pose substantial challenges to the reliability and trustworthiness of object detectors [16,17]. Effectively addressing these uncertainties is essential for ensuring the practical applicability, trustworthiness, and robustness of object detectors in real-world scenarios. However, conventional evaluation metrics such as precision, recall, F1-score, and average precision (AP) often fail to characterize or quantify the impact of uncertainty adequately. This limitation has led to a growing research interest in quantifying uncertainty in object detection models and the broader field of machine learning [18].

Numerous techniques for uncertainty quantification in object detection have been developed to enhance the reliability of prediction outputs. Among the most prominent are Bayesian neural networks [19], Monte Carlo (MC) dropout [20,21], Platt and temperature scaling [22], Deep Ensembles [23], variational inference [24], and evidential deep learning (EDL) [25]. While these methods demonstrate substantial utility in capturing uncertainties in object detection models, they are not without limitations.

Methods such as MC-Dropout and Deep Ensembles incur substantial computational overhead. Their reliance on multiple forward passes increases inference time and computational cost, rendering them impractical for resource-constrained environments like edge devices and real-time systems. Similarly, Bayesian neural networks and variational inference introduce additional complexity due to their mathematically intricate formulations, often requiring careful tuning and expertise in implementation, which poses barriers to widespread adoption and scalability in applied settings. While computationally lighter, Platt and temperature scaling merely adjust confidence scores; they do not guarantee statistically valid calibrated probabilities or alter the original predicted class. Moreover, EDL requires architectural modifications, further complicating deployment.

Recently, conformal prediction has emerged as a robust framework for uncertainty quantification in machine learning, distinguished by its model-agnostic nature [26,27]. Unlike ensemble methods, which require training multiple models [28], or EDL, which necessitates modified loss functions [29], conformal prediction imposes no architectural constraints, ensuring compatibility with any model or dataset. Furthermore, compared with calibration techniques such as Platt scaling and temperature scaling, which merely adjust classification probabilities and lack formal coverage guarantees [22], conformal prediction provides rigorous finite-sample coverage guarantees for both classification and regression tasks by operating on the output of a pretrained model. This simplicity and flexibility make conformal prediction particularly effective in resource-constrained settings, such as edge devices for real-time object detection, where computational overhead from methods like Bayesian neural networks or MC-Dropout poses challenges [20]. By providing a guaranteed coverage framework for uncertainty estimation, conformal prediction directly addresses limitations of existing techniques, thereby enhancing reliability in complex safety-critical applications.

This versatility of conformal prediction is particularly relevant to object detection, where it can address uncertainty across distinct subtasks. Object detection combines localization (a regression task predicting object bounding boxes) with classification (assigning semantic labels). In safety-critical applications such as pedestrian localization in autonomous vehicles [30], railway signaling [31], and delineating regions of interest in medical radiography [32], managing spatial uncertainty is paramount. In contrast, semantic accuracy takes precedence in domains like parasitological analysis, where detecting and accurately counting parasite eggs in stool samples directly informs treatment decisions, rendering correct class-label assignment more critical than precise localization [33,34]. These examples highlight how the relative importance of spatial versus semantic uncertainty varies considerably across application domains. Although conformal prediction has been widely employed to establish tolerance margins for spatial localization, its application to ensure valid coverage of true class labels in the semantic subtask remains, to the best of our knowledge, significantly underexplored.

The limited exploration of conformal prediction for the semantic aspect of object detection is, in part, due to the design of current object detection models. At inference time, these models typically output a confidence score only for the top-ranked class, without providing the full confidence distribution over all classes. This lack of per-class confidence scores fundamentally limits the applicability of conformal prediction in the semantic subtask, as these scores are essential for constructing valid prediction sets. To address this critical limitation, we introduce a conformal prediction framework—specifically, inductive conformal prediction (ICP)—tailored for object detection that ensures valid class-label coverage. ICP is a variant of conformal prediction that uses a dedicated calibration set to provide statistically valid coverage of class labels. By constructing and using multiclass confidence distributions, our approach enhances the semantic reliability of object detectors and improves their trustworthiness in high-stakes domains where classification accuracy is paramount.

The key contributions of the paper can be summarized as follows:

- *Introducing the application of ICP for semantic classification in object detection*: Whereas previous work focused solely on bounding-box localization guarantees, we tackle the unaddressed challenge of achieving guarantees for class-label coverage of detected objects, by designing an application of ICP for semantic classification within object detection pipelines, distinguishing our work from existing conformal prediction applications.
- *Enabling conformal prediction in standard object detectors via score adaptation*: We address a key practical barrier by successfully adapting widely used object detection models (Faster R-CNN, RetinaNet, YOLO11, RT-DETR) to expose the necessary per-class confidence scores, including a heuristic for estimating the background class confidence. This modification overcomes a critical limitation of standard models, which typically output only the top-class score, thereby enabling conformal prediction for valid class-wise coverage in the semantic subtask of object detection.
- *Curating and introducing MS-COCO-20 as a benchmark subset*: We introduce MS-COCO-20, a curated 20-class subset derived from MS-COCO, designed to serve as an efficient (in terms of computational cost and experimentation time) and accessible benchmark for evaluating and comparing nonconformity scores for the classification aspect of object detection.
- *Empirical validation of guaranteed coverage on the MS-COCO-20 benchmark and two use-case datasets*: We empirically validate the effectiveness of our conformal prediction approach on the MS-COCO-20 benchmark, as well as on two use-case datasets focused on detecting parasite eggs in microscopy images. Our results demonstrate that finite-sample guaranteed class-label coverage can be achieved for detected instances in both general-purpose and practical, specialized settings, highlighting the potential of conformal prediction to enhance trustworthiness in downstream analysis.

## 2. Materials and Methods

**Figure 1:**
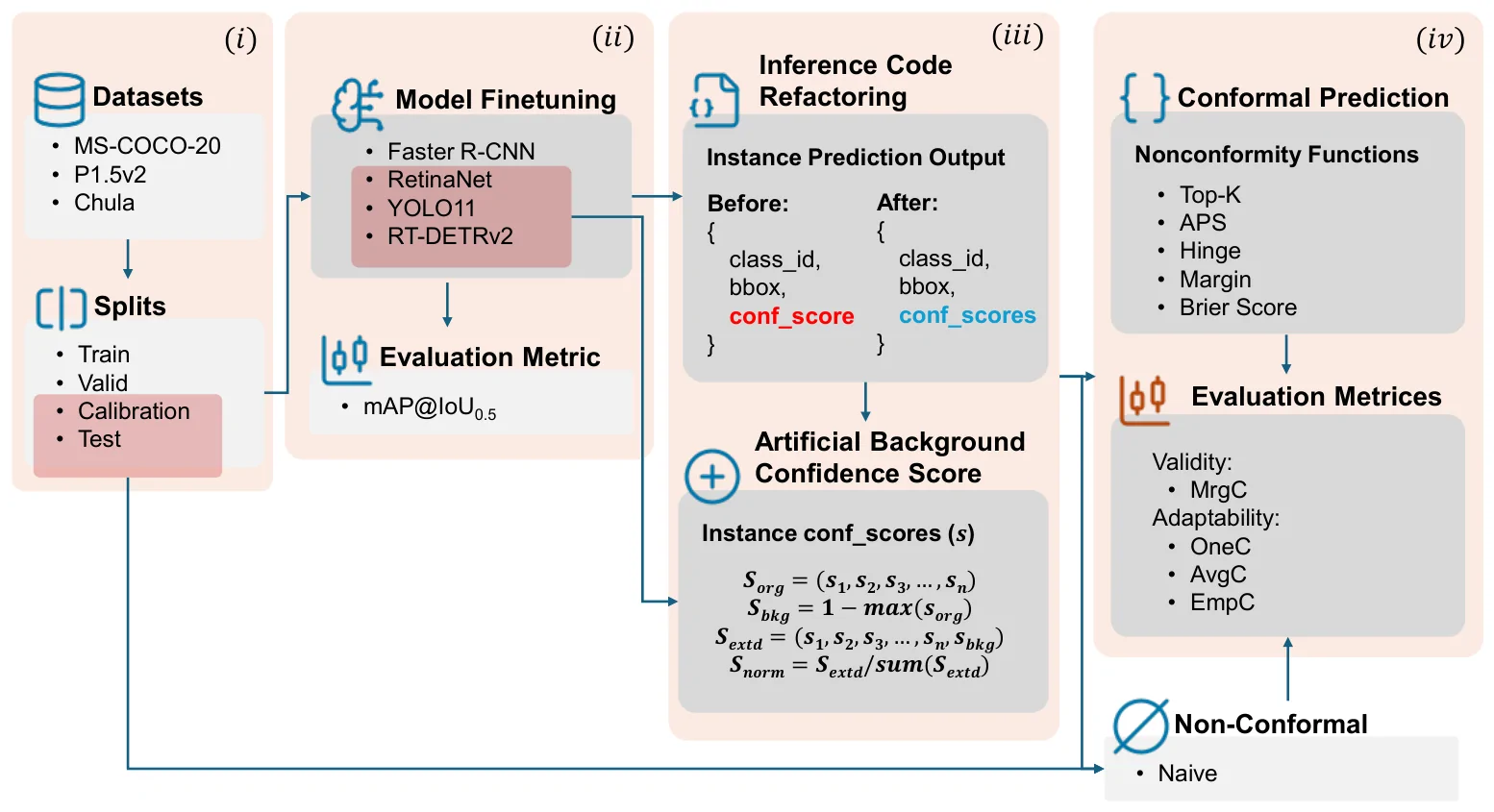
An overview of our workflow for generating class-label conformal prediction sets with statistical guarantees in object detection. The process involves (i) preparing the data using a dedicated calibration set; (ii) fine-tuning standard baseline object detectors; (iii) refactoring model outputs into confidence score distributions, appending a heuristic artificial background class (where applicable), and normalizing the result; and (iv) applying conformal prediction to produce prediction sets that are guaranteed to contain the true class label at a user-specified significance level (e.g., α=0.1). Icons from the Lucide project (https://lucide.dev/, accessed on 20 July 2026), licensed under the ISC License © Lucide Contributors.

This section details the methodology used to develop and evaluate a conformal prediction approach for generating object detection predictions with class-label guarantees. Figure 1 provides a schematic overview of our four-stage process. The four-stage pipeline involves (i) preparing datasets, including a dedicated calibration split; (ii) fine-tuning and evaluating baseline object detection models; (iii) refactoring model outputs into normalized confidence distributions, augmented with a heuristic artificial background class (except Faster R-CNN); and (iv) applying nonconformity functions to construct and evaluate statistically valid prediction sets.

### 2.1. Datasets

To facilitate consistent experimentation and establish a benchmark for evaluating the effectiveness of conformal prediction in providing guaranteed class-label coverage in object detection, we introduce MS-COCO-20. This dataset, derived from the 80-class MS-COCO dataset [35], comprises the 20 most frequent object classes in the original MS-COCO validation set, as determined by instance frequency. Selecting 20 classes strikes a balance between computational efficiency and semantic diversity, enabling faster experimentation without sacrificing generalizability. By focusing on the most populated classes, we ensure sufficient calibration and evaluation samples per class, which is critical for reliable uncertainty quantification. Importantly, this frequency-based selection does not affect the validity of ICP’s coverage guarantee, which holds for any class composition provided the calibration and test detections are exchangeable. The MS-COCO-20 dataset consists of images from the original MS-COCO training and validation splits that contain at least one instance of the selected object classes. The class distribution within MS-COCO-20 is shown in Table 1. Baseline models were trained and validated using the original MS-COCO training split. For the conformal prediction experiments, the calibration and test sets were sampled from the original MS-COCO validation set. We aimed for a 50/50 split; however, the co-occurrence of multiple object classes within the same image prevented an exact 50/50 split. This approximate 50/50 partition was selected because it ensures balance: neither the calibration nor the test set is so small as to cause either quantile instability or high-variance evaluation.

To further evaluate conformal prediction for object detection, we also selected two publicly available use-case-specific datasets focusing on parasite egg detection. The first dataset, AI4NTD KK2.0 P1.5 STH & SCHm v2 [36], hereafter referred to as P1.5v2, contains four types of parasite eggs (Table 2). For P1.5v2, the training and validation sets were generated from the original training data at approximately a 4:1 ratio. The original validation and test sets were used to generate the calibration and final test sets, respectively. The second dataset, Chula-ParasiteEgg-11 (Chula) [37], comprises 11 parasite egg classes, with its class distribution across splits outlined in Table 3. Training and validation sets were generated from the original training data, and calibration and test sets were obtained from the original test data.

### 2.2. Object Detection Models

To evaluate the effectiveness of conformal prediction in providing guaranteed class-label coverage across diverse object detection architectures, we selected four established models (see Table 4): Faster R-CNN, RetinaNet, YOLO11, and RT-DETRv2. This selection encompasses both established Deep Convolutional Neural Networks (DCNNs) and emerging Transformer-based paradigms, enabling us to assess the robustness of conformal prediction across diverse architectures.

- **Faster R-CNN** [38]: An established two-stage detector, included due to foundational role and widespread adoption.
- **RetinaNet** [39]: A one-stage detector chosen for its use of focal loss, a key contribution to handling class imbalance in real-world object detection scenarios.
- **YOLO11** [40]: A one-stage detector known for its efficient architecture, enabling fast inference speeds crucial for real-time applications.
- **RT-DETRv2** [41]: A one-stage detector that leverages self-attention mechanisms in its Transformer-based architecture to improve long-range dependency modeling.

A significant challenge in applying conformal prediction to object detection models stems from limited access to per-class confidence scores during inference, as commonly used codebases—primarily Detectron2 [42] and Ultralytics [43]—typically return only the confidence score for the predicted class. However, most conformal prediction approaches for classification tasks require softmax probability outputs (or equivalent sigmoid-weighted confidence scores) for all classes of a given instance (including background) to compute nonconformity scores. To address this, we refactored the inference process in each model codebase to extract all per-class confidence scores without altering the training pipeline or compromising the model-agnostic nature of conformal prediction. Crucially, this constitutes an inference-only code change; no model layers, weights, or training procedures were modified. To understand the practical implications, we next examine the output categories of object detection models and their relevance to conformal prediction.

When evaluating object detection models, three primary outcomes are considered: True Positives (correctly identified objects), False Positives (detections that do not correspond to a ground-truth object, including those in background regions), and False Negatives (missed objects). In the context of conformal prediction applied to object detection, per-class confidence scores are essential for constructing prediction sets for detected objects, namely, True Positives and False Positives. These scores allow conformal prediction to estimate the uncertainty associated with each predicted instance and determine whether the actual class label is covered. In contrast, False Negatives correspond to objects that the model failed to detect. Because conformal prediction operates on model outputs, it cannot correct for or quantify errors arising from missed detections. Consequently, False Negatives reflect intrinsic limitations of the base detector rather than a shortcoming of the conformal prediction framework; thus, they lie outside the scope of conformal coverage guarantees. To minimize their occurrence, we set a deliberately low detection confidence threshold of 0.1, maximizing recall over precision. Crucially, although object detection models typically provide only confidence scores for foreground classes associated with detected objects, the availability and quality of confidence scores, particularly for a dedicated background class, vary substantially across architectures. This inconsistency necessitates additional adaptations to enable a universally applicable conformal prediction framework.

Handling of background classes varies across object detection architectures. Faster R-CNN, for instance, incorporates a background class via softmax activation in its classification head, differentiating non-object regions. In contrast, models such as RetinaNet, YOLO11, and RT-DETRv2 output independent sigmoid-weighted per-class confidence scores and, therefore, lack an explicit background class. This architectural difference reflects deliberate design choices. Faster R-CNN’s two-stage (“crop-and-classify” design) pipeline assumes one object per region proposal (mutual exclusivity), making softmax the natural choice, whereas the rest of the one-stage detectors treat classification as independent binary decisions to handle potentially overlapping labels that are better served by sigmoid activations. The nonconformity score constructions used in this study operate on a score vector on the probability simplex (non-negative entries summing to one), while raw sigmoid outputs are not normalized in this way. To make sigmoid-based detectors compatible with a unified conformal scoring pipeline, we introduce an artificial background confidence score for models without a native background class (Algorithm 1) as $1-\max(s_{\mathrm{orig}})$ and then normalize the extended score vector. We treat this as a pragmatic score-adaptation heuristic for implementation compatibility, not as a new probabilistic model or a theoretical requirement for ICP validity.
**Algorithm 1** Background Confidence Score and Normalization  1:**procedure** ComputeNormalizedScores($s_{\mathrm{orig}}$) ▹$s_{\mathrm{orig}}=\left(s_{1},\ldots ,s_{n}\right)$ are the original foreground confidence scores**Require:** Original foreground confidence scores $s_{\mathrm{orig}}$**Ensure:** Normalized confidence scores $s_{\mathrm{norm}}$ including background class  2:    $s_{\mathrm{background}}\leftarrow 1-\max\left(s_{\mathrm{orig}}\right)$  3:    $s_{\mathrm{extended}}\leftarrow \left(s_{1},\ldots ,s_{n},s_{\mathrm{background}}\right)$  4:    $\mathrm{sum}\_\mathrm{val}\leftarrow \sum s_{\mathrm{extended}}$  5:    $s_{\mathrm{norm}}\leftarrow s_{\mathrm{extended}}/\mathrm{sum}\_\mathrm{val}$              ▹ Element-wise division  6:    **return** $s_{\mathrm{norm}}$  7:**end procedure**

### 2.3. Conformal Prediction

Conformal prediction is a general framework, not a specific algorithm, designed to produce valid prediction regions with statistical guarantees [27]. Specifically, conformal prediction probabilistically ensures that the actual outcome lies within the prediction region with a user-specified confidence level of at least 1−α, where α∈(0,1) is the significance level controlling the allowable error rate. This probabilistic guarantee is marginal coverage, meaning that it holds on average over the joint distribution of the data and model predictions, rather than conditioning on specific inputs. The conformal prediction framework is model-agnostic and can be applied on top of any underlying predictive model, making it especially valuable for complex tasks such as object detection, which require reliable spatial and semantic coverage and, thus, uncertainty quantification in both dimensions.

Inductive conformal prediction (ICP) [27], also known as split conformal prediction, is a practical variant of conformal prediction that improves computational efficiency by partitioning the available data into a proper training set $Z^{\mathrm{train}}$ (used for baseline model training) and a separate calibration set $Z^{\mathrm{Cal}}$. The calibration set is used to create nonconformity scores, which, in turn, are used to construct valid prediction sets for new examples. A new test dataset $Z^{\mathrm{test}}$, comprising examples not included in either the proper training or calibration datasets, is assumed to be independently and identically distributed (i.i.d.) with $Z^{\mathrm{cal}}$. This i.i.d. assumption between $Z^{\mathrm{cal}}$ and $Z^{\mathrm{test}}$ is critical for the validity of conformal prediction. Notably, the $Z^{\mathrm{train}}$ may or may not be i.i.d. with $Z^{\mathrm{cal}}$ and $Z^{\mathrm{test}}$. Compared with transductive conformal prediction, which requires re-evaluating the model for each new test point and class label, ICP provides the same marginal coverage guarantees with significantly less computational overhead. Here, we outline the formulation of conformal prediction to ensure guarantees for class-label coverage in object detection under this i.i.d. assumption [26].

The ICP guarantee in this work is marginal at the detection level. It holds on average over all predicted detections, not on a per-image basis. Calibration and inference are performed per detection (post-NMS), using only per-class confidence vector each detection, and exchangeability is assumed over these pooled per-detection nonconformity scores. Marginal detection-level ICP provides a provable, distribution-free finite-sample guarantee; exact per-image or per-class conditional guarantees are distribution-free impossible for finite samples and require impractical sample sizes or conservative bounds. For detection *i* with set ${\hat{C}}_{i}^{\alpha }$, standard ICP yields

$$P\;\left(y_{i}\in {\hat{C}}_{i}^{\alpha }\right)\geq 1-\alpha ,$$

assuming exchangeability between calibration and test detections.

After training the baseline model, we utilize the calibration ($Z^{\mathrm{cal}}$) and test ($Z^{\mathrm{test}}$) sets to implement conformal prediction. In ICP, the goal is to construct a prediction set that, for each class, contains the true class at least 1−α of the time, probabilistically. To do so, we compute nonconformity scores from the per-class confidence scores of the models, which form a probability distribution over all foreground classes and a background class. When the model lacks an explicit background class, an artificial score is introduced, and the complete set of scores is normalized (Algorithm 1). From $Z^{\mathrm{cal}}$, we estimate the empirical (1−α) quantile of these nonconformity scores. Then, for each test example in $Z^{\mathrm{test}}$, we construct the prediction set by including every class whose score is above this threshold. This procedure guarantees marginal coverage at level 1−α.

#### 2.3.1. Nonconformity Scores

Nonconformity functions (NCFs) are central to conformal prediction, quantifying the “strangeness” or “nonconformity” of a data point with respect to its label. A higher nonconformity score indicates a poorer fit. For a given vector of confidence scores s and true label *y*, a model-agnostic NCF *A* computes a nonconformity score ρ=A(s,y).

In the calibration phase, nonconformity scores $\rho_{i}=A\left(s_{i},y_{i}\right)$ are computed for each $\left(s_{i},y_{i}\right)$ pair in the calibration set $Z^{\mathrm{cal}}$. For a new test example with confidence score vector $s_{t}$, a nonconformity score $\rho_{t}^{y}=A\left(s_{t},y\right)$ is calculated for each possible candidate label y∈Y. Subsequently, a *p*-value $p_{t}^{y}$ is derived for each candidate label *y* by comparing its nonconformity score $\rho_{t}^{y}$ to the distribution of nonconformity scores from the calibration set:

$$p_{t}^{y}=\frac{\left|\left\{i\in Z^{\mathrm{cal}}:\rho_{i}\geq \rho_{t}^{y}\right\}\right|+1}{m+1}$$

where $m=|Z^{\mathrm{cal}}|$ is the calibration set size.

Finally, the prediction set ${\hat{C}}_{t}^{\alpha }$ for the test example includes all candidate labels *y* whose *p*-value is greater than or equal to the significance level α:

$${\hat{C}}_{t}^{\alpha }=\left\{y:p_{t}^{y}\geq \alpha \right\}.$$

This construction gives us the probabilistic marginal coverage guarantee; i.e., the true label is included in the prediction set with at least 1−α marginal probability. This finite-sample coverage guarantee hinges on the i.i.d. property between the calibration and test sets.

In this research, we employed five distinct nonconformity scores, elaborated as follows. These nonconformity scores were applied to per-class confidence scores from adapted models, incorporating the artificial background score as described in Section 2.2 and Algorithm 1.

##### Top-K Method

The Top-K nonconformity score quantifies nonconformity based on the confidence score rank of the true label. For the *i*th instance $\left(x_{i},y_{i}\right)$ from the calibration set, model confidence scores $s_{i}=s_{i,j}$ (where *j* indexes the classes) are sorted in descending order. The nonconformity score $\rho_{i}$ for this *i*th instance is the rank (position) of the true label $y_{i}$ within this sorted list [44]. While typically applied to softmax probabilities in multiclass classification, we adapted this method to utilize normalized confidence scores. For models without a native background confidence score, these scores were generated as detailed in Algorithm 1.

##### Adaptive Prediction Sets (APS) Method

The APS method defines nonconformity based on the cumulative confidence score required to include the true label. For the $i^{th}$ calibration instance $\left(x_{i},y_{i}\right)$, the confidence scores $s_{i}=\left\{s_{i,j}\right\}$ are sorted in descending order. The nonconformity score $\rho_{i}$ for this *i*th instance is the sum of scores from the top-ranked class down to the rank where the true label $y_{i}$ appears. These nonconformity scores are used in the calibration phase to determine a threshold $\hat{\tau }$ corresponding to the desired coverage level 1−α. During inference, for a new instance, classes are added to the prediction set in descending order of score until their cumulative sum reaches $\hat{\tau }$, resulting in adaptively sized sets [45].

##### Hinge Method

The Hinge nonconformity score directly uses the confidence score of the model in the true label. For the $i^{th}$ calibration instance $\left(x_{i},y_{i}\right)$, the score is calculated as $\rho_{i}=1-s_{i,y_{i}}$, where $s_{i,y_{i}}$ is the confidence score for the true class-label $y_{i}$. These scores, $\rho_{i}$, are used during calibration to determine a threshold, $\rho^{*}$, that ensures the 1−α coverage guarantee. During inference, for the scores $s_{\mathrm{new}}$ of a new instance, any candidate class *y* with nonconformity score $1-s_{\mathrm{new},y}\leq \rho^{*}$ is included in the prediction set.

##### Margin Method

This method defines nonconformity based on the margin between the confidence score of the true label and the highest confidence of any other class. For the *i*th calibration instance $\left(x_{i},y_{i}\right)$, the nonconformity score $\rho_{i}$ is calculated as

$$\rho_{i}=\max_{k\neq y_{i}}s_{i,k}-s_{i,y_{i}}$$

where $s_{i,k}$ is the confidence score for class *k* for instance *i*. A smaller (or more negative) $\rho_{i}$ indicates better separation and, thus, higher conformity. Calibration involves sorting these $\rho_{i}$ scores to find a threshold $\rho^{*}$ for the 1−α coverage level. During inference, for a new instance, a candidate class *y* is included in the prediction set if its calculated margin score satisfies the following inequality:

$$\max_{k\neq y}s_{\mathrm{new},k}-s_{\mathrm{new},y}\leq \rho^{*}$$

##### Brier Score Method

This nonconformity score leverages the Brier score to measure discrepancy between confidence scores and the true outcome. For the *i*th calibration instance $\left(x_{i},y_{i}\right)$, the nonconformity score $\rho_{i}$ is computed as the mean squared error between the confidence score vector $s_{i}={\left\{s_{i,j}\right\}}_{j=1}^{K}$ and a one-hot encoded vector for the true label $y_{i}$. Explicitly,

$$\rho_{i}=\frac{1}{K}\sum_{j=1}^{K}{\left(I\left(j=y_{i}\right)-s_{i,j}\right)}^{2}$$

where *j* indexes classes from 1 to *K*, $s_{i,j}$ is the confidence score for class *j* on instance *i*, and $I(j=y_{i})$ is 1 if $j=y_{i}$ and 0 otherwise. A smaller $\rho_{i}$ signifies a better prediction (closer to one-hot truth) and, thus, higher conformity. These scores are calibrated by sorting them to find a threshold $\rho^{*}$ ensuring 1−α coverage. During inference, for a new instance, a potential nonconformity score $\rho_{\mathrm{new}}\left(y\right)$ is calculated for each candidate class *y* (treating *y* as the hypothetical true label) using the same Brier formula:

$$\rho_{\mathrm{new}}\left(y\right)=\frac{1}{K}\sum_{j=1}^{K}{\left(I\left(j=y\right)-s_{\mathrm{new},j}\right)}^{2}.$$

Class *y* is then included in the final prediction set if its potential nonconformity score $\rho_{\mathrm{new}}\left(y\right)\leq \rho^{*}$.

It is necessary to remember that all nonconformity functions evaluated in this work were utilized strictly to measure the nonconformity of the confidence scores of the detector. They served no auxiliary probabilistic calibration or modeling role within our pipeline. In addition to five distinct nonconformity scores, we applied a Naive approach, which can be viewed as a non-conformal version of the APS approach.

##### Naive Method

This method constructs prediction sets by directly thresholding cumulative confidence scores. For the $i^{th}$ instance $x_{i}$, its confidence scores $s_{i}=s_{i,j}$ (where *j* indexes classes) are sorted in descending order. Classes are then added to the prediction set $\hat{C}$ in decreasing score until their cumulative sum reaches a predefined threshold, typically 1−α. Unlike conformal methods, this approach uses a fixed threshold directly, without a true-label-based calibration step.

### 2.4. Computational Overhead

During the calibration phase, all nonconformity methods operate post hoc on the class score vectors generated via a single forward pass over the *K* classes per instance, allowing the calibration quantile $\hat{q}$ to be computed entirely offline. Consequently, runtime deployment is highly optimized, leaving only the inference-stage nonconformity score calculation and a simple scalar threshold comparison against $\hat{q}$. Theoretically, during inference, Top-K, APS, and Naive are computationally efficient, requiring a single sorting operation or a localized vector mask over the post-threshold detections, thereby minimizing overhead in high-density scenes. Conversely, Hinge, Margin, and Brier require more computational resources in dense environments; for each candidate proposal, these functions perform element-wise tensor subtractions or squaring operations across the entire *K*-dimensional confidence score (probability) array, so their latency scales with proposal density.

### 2.5. Evaluation Metrics

The effectiveness of conformal prediction was evaluated using several key metrics that assess both the validity (coverage) and efficiency (set size) of the generated prediction sets ${\hat{C}}_{i}$.

#### 2.5.1. Coverage-Related Metrics

##### Marginal Coverage (MrgC)

This primary validity metric measures the proportion of instances where the true class-label $y_{i}$ is included in its corresponding prediction set ${\hat{C}}_{i}$ across the entire test dataset of size *N*. It estimates marginal probability. The target for conformal prediction is ≥1−α.

$$\mathrm{MrgC}=\frac{1}{N}\sum_{i=1}^{N}I\left(y_{i}\in {\hat{C}}_{i}\right)$$

where I(·) is the indicator function.

#### 2.5.2. Efficiency-Related Metric

##### Average Set Size (AvgC)

This metric measures the average number of classes in the prediction sets, indicating the average level of prediction ambiguity or inefficiency. Smaller values are preferred, provided the coverage target is met.

$$\mathrm{AvgC}=\frac{1}{N}\sum_{i=1}^{N}\left|{\hat{C}}_{i}\right|$$

where |·| denotes the cardinality (size) of the set.

##### Singleton Rate (OneC)

This measures the fraction of prediction sets containing exactly one class label $\left(|{\hat{C}}_{i}|=1\right)$. A higher rate, combined with high coverage, suggests that the model makes precise, confident predictions.

$$\mathrm{OneC}=\frac{1}{N}\sum_{i=1}^{N}I(|{\hat{C}}_{i}\left|=1\right)$$

##### Empty Set Rate (EmpC)

Particularly relevant for object detection, this metric calculates the fraction of predictions for which the prediction set is empty $\left(|{\hat{C}}_{i}|=0\right)$. An ideal conformal procedure should avoid generating empty sets for relevant objects in i.i.d. scenarios. However, this metric can also be informative in situations such as out-of-distribution (OOD) detection.

$$\mathrm{EmpC}=\frac{1}{N}\sum_{i=1}^{N}I(|{\hat{C}}_{i}\left|=0\right)$$

## 3. Results and Discussion

### 3.1. Effectiveness of the Baseline Models

**Figure 2:**
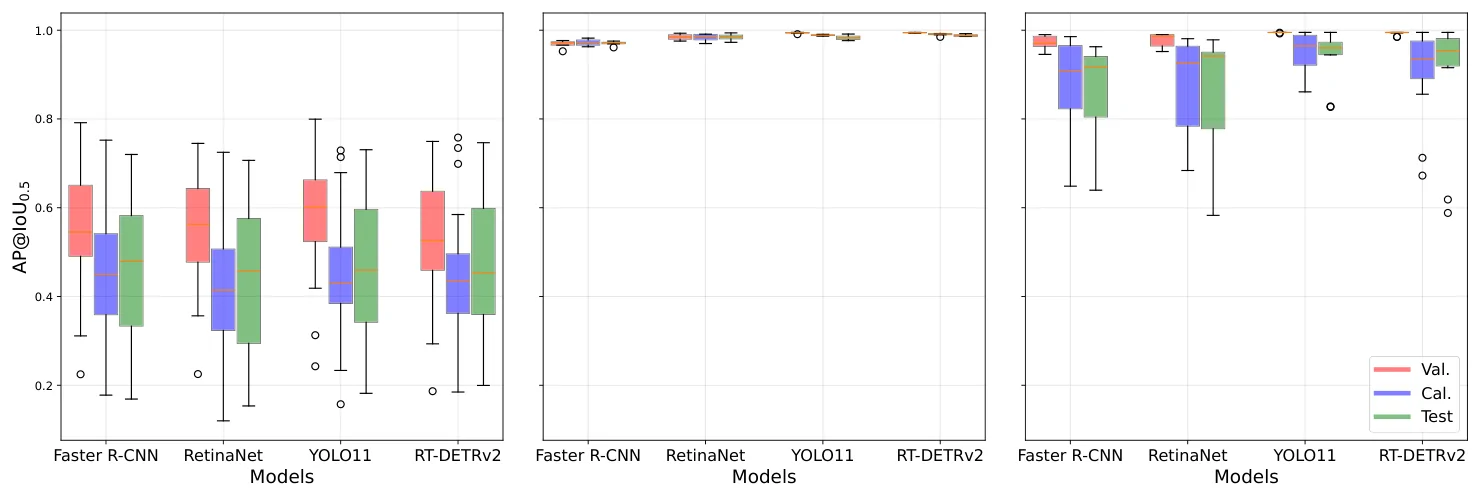
Predictive performance of baseline models on evaluation splits. Box plots show the distribution of ${\mathrm{AP}@\mathrm{IoU}}_{0.5}$ values across classes for the validation (Val.), calibration (Cal.), and test sets for four object detection models on three datasets: MS-COCO-20 (**left**), P1.5 (**middle**), and Chula (**right**).

Figure 2 presents the predictive performance of the four baseline object detection models (Faster R-CNN, RetinaNet, YOLO11, RT-DETRv2) across the three datasets (MS-COCO-20, P1.5v2, Chula) on the validation, calibration, and test splits. The metric shown is ${\mathrm{AP}@\mathrm{IoU}}_{0.5}$, visualized as box plots showing the performance distribution across classes within each split. ${\mathrm{AP}@\mathrm{IoU}}_{0.5}$ denotes the area under the precision–recall curve for a specific class, considering predictions with an IoU of at least 50% as correct detections. Here, AP@IoU_0.5_ is reported as the commonly used baseline detector metric to contextualize detector quality before conformalization, while the main objective of this manuscript is ICP evaluation through conformal metrics (MrgC, AvgC, OneC, EmpC). For reference, AP@IoU_0.5:0.95_ and Recall@IoU_0.5_ are reported in the Appendix A.

The results highlight substantial differences in task difficulty across datasets. On the left panel (MS-COCO-20 dataset), we observe moderate ${\mathrm{AP}@\mathrm{IoU}}_{0.5}$ values, with medians typically ranging between approximately 0.40 and 0.50 depending on the model and split. The box plots exhibit considerable spread and outliers, indicating notable variability in effectiveness across the 20 classes. Furthermore, noticeable differences are observed among the validation, calibration, and test splits for some models, suggesting potential shifts in class-wise effectiveness. All four models demonstrate broadly similar profiles on this dataset.

In contrast, the middle panel (P1.5v2 dataset) illustrates a substantially straightforward task for the evaluated models. All models achieve very high ${\mathrm{AP}@\mathrm{IoU}}_{0.5}$ values, with medians consistently above 0.95. As shown in Figure 2, the box plots are incredibly narrow, with low variance and few outliers, indicating highly consistent performance across all four parasite egg classes. Crucially, the effectiveness distributions across the validation, calibration, and test splits are remarkably similar and tightly clustered across all models, indicating a high degree of consistency among these data partitions in terms of model predictive performance.

The right panel (Chula dataset) presents an intermediate scenario. ${\mathrm{AP}@\mathrm{IoU}}_{0.5}$ values are generally high, with medians for YOLO11 and RT-DETRv2 above 0.9. At the same time, Faster R-CNN and RetinaNet show slightly lower medians, particularly on the calibration and test splits. The range and interquartile range are larger than on P1.5v2, and outliers are present. While effectiveness is generally substantial, slight variations across the validation, calibration, and test splits are observed, particularly for Faster R-CNN and RetinaNet. More recent models, YOLO11 and RT-DETRv2, appear to exhibit slightly more stable performance across splits on this dataset than older architectures.

These baseline effectiveness results are critical for interpreting subsequent conformal prediction outcomes. Datasets such as P1.5v2, in which models achieve high, stable effectiveness across splits, represent an ideal scenario for conformal prediction, as calibration-set nonconformity scores are likely highly representative of those on the test set. Conversely, datasets such as MS-COCO-20—where baseline effectiveness is lower and more variable, and where effectiveness distributions show some divergence across splits—pose a more challenging setting for achieving tight, reliable coverage guarantees, highlighting the importance of robust nonconformity score definitions and sufficient calibration data. The Chula dataset represents a scenario in which the task is manageable, but potential split differences may still affect the performance of conformal prediction. The observed differences in stability across models on Chula also suggest that the choice of the underlying base detector may influence the properties of the resulting prediction sets.

### 3.2. Impact of Artificial Background Confidence Score

**Figure 3:**
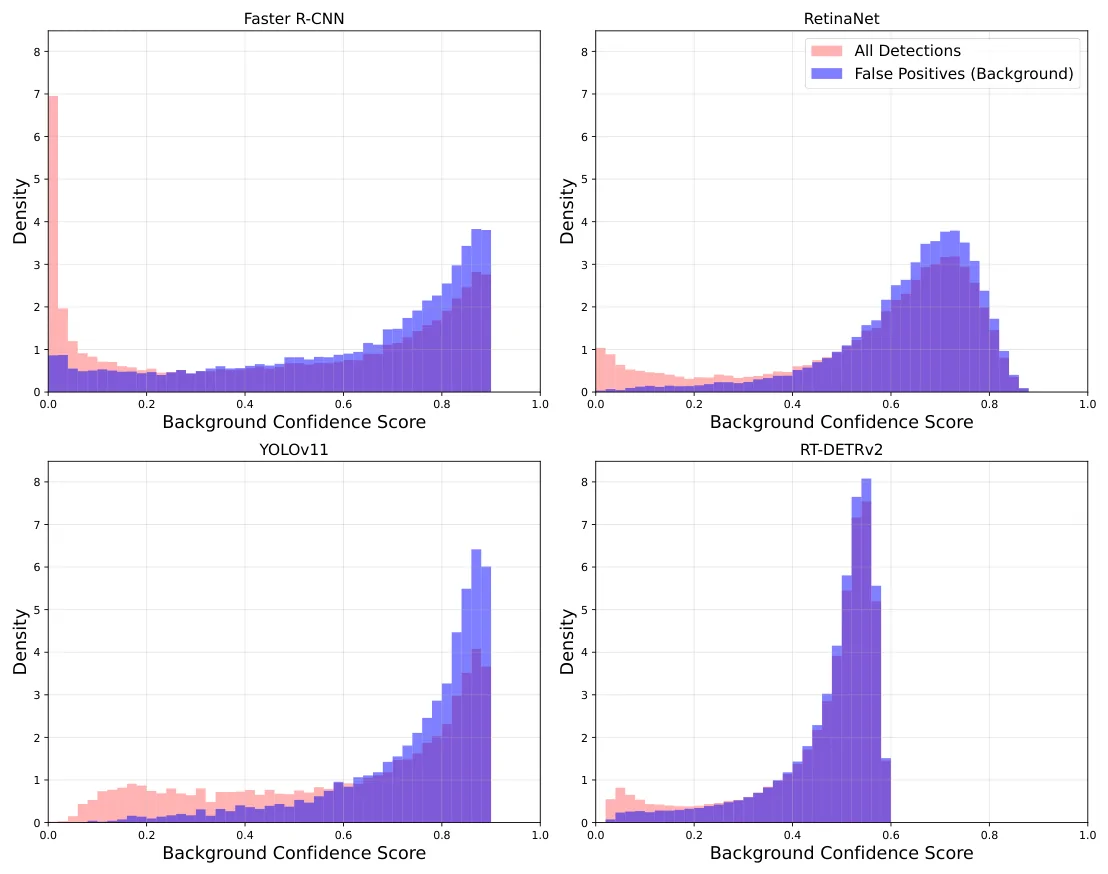
Distribution of background confidence scores on the MS-COCO-20 test set for Faster R-CNN, RetinaNet, YOLO11, and RT-DETRv2. Overlaid histograms show “All Detections” and “False Positives (Background)”.

Figure 3 illustrates the effectiveness of the proposed artificial background confidence score in quantifying detected background instances (False Positives). Analysis was conducted on the challenging MS-COCO-20 test split, which, as discussed in Section 2, represents a more complex scenario than other datasets. Overlaid histograms reveal a consistent pattern across all evaluated models: the background confidence score distribution for all detections is bimodal; foreground objects tend to yield low background confidence scores, whereas False Positives (background regions misclassified as foreground) are overwhelmingly concentrated at high background confidence scores. This demonstrates that, whether native (Faster R-CNN) or artificially computed (RetinaNet, YOLO11, RT-DETRv2), the background score effectively assigns high confidence to true background detections. This consistent behavior observed on the complex MS-COCO-20 dataset validates the heuristic approach as a practical and valuable method for estimating background confidence scores for models lacking a native background class, thereby enabling conformal prediction across diverse object detection architectures. Similar distributions for the P1.5v2 and Chula datasets are presented in Appendix B.

### 3.3. Effectiveness of the Conformal Prediction Nonconformity Functions

The evaluation of conformal prediction methods is based on two core principles: validity and adaptivity [26]. Validity refers to the ability of a process to consistently achieve the desired marginal coverage guarantee (1−α), while adaptivity describes its capacity to produce prediction sets whose size reflects individual instance difficulty, yielding smaller, more informative sets for straightforward inputs and larger sets for ambiguous ones.

**Figure 4:**
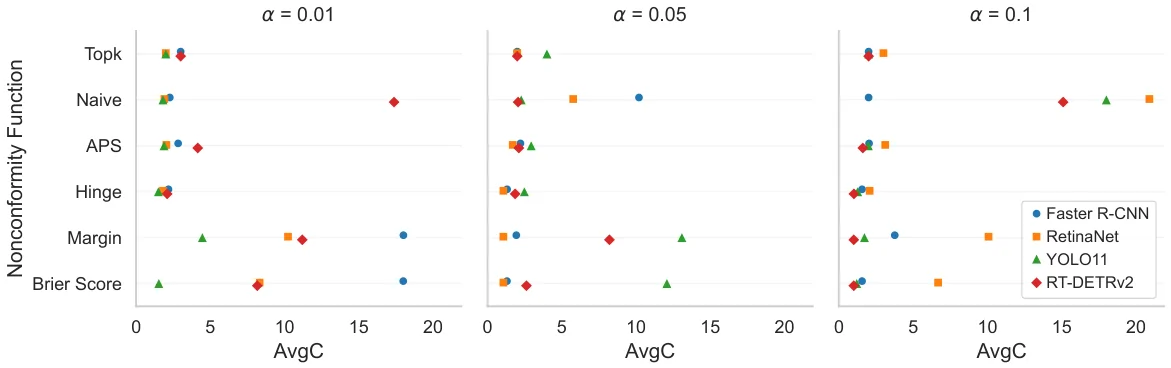
Empirical AvgC for different NFCs and base models on the MS-COCO-20 dataset. Results are shown for target significance levels (α∈{0.01,0.05,0.1}). Lower AvgC signifies more efficient prediction sets.

**Figure 5:**
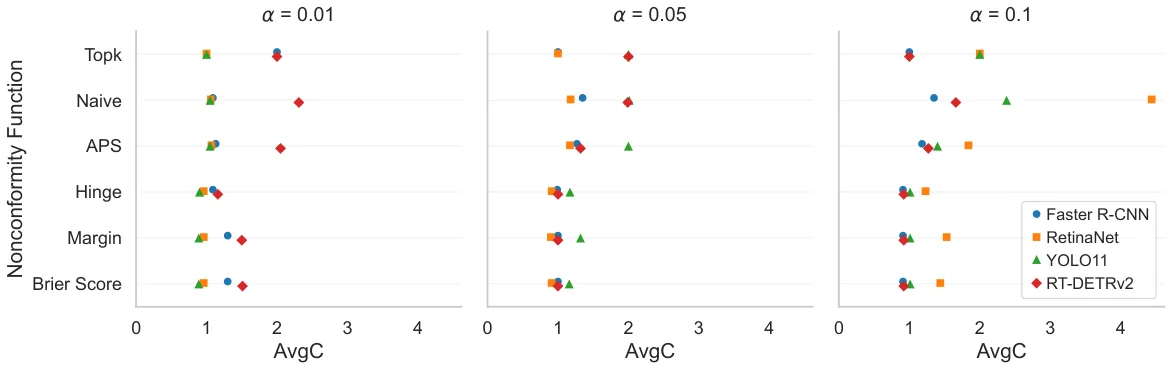
Empirical AvgC for different NCFs and base models on the P1.5 dataset. Results are shown for target significance levels (α∈{0.01,0.05,0.1}). Lower AvgC signifies more efficient prediction sets.

**Figure 6:**
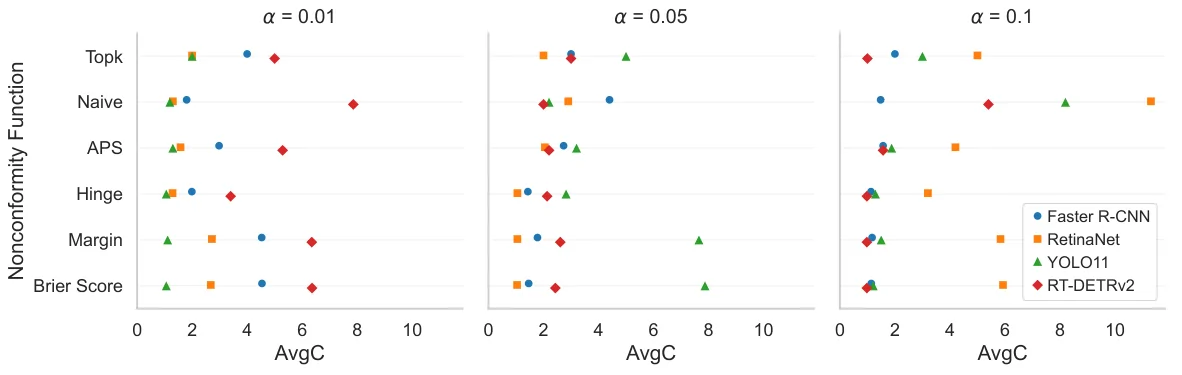
Empirical AvgC for different nonconformity scores and base models on the Chula-ParasiteEgg11 dataset. Results are shown for target significance levels (α∈{0.01,0.05,0.1}). Lower AvgC signifies more efficient prediction sets.

We evaluated the effectiveness of the implemented nonconformity scores (Top-K, APS, Hinge, Margin, Brier score) and the non-conformal prediction method (Naive), as introduced in Section 2.3.1, with respect to the metrics introduced in Section 2.5. The evaluation was performed across all four fine-tuned object detection models (see Section 3.1) and across the three datasets (see Section 2.1), for different significance levels α. Detailed results are presented in Table 5, Table 6 and Table 7 for the MS-COCO-20, P1.5v2, and Chula datasets, respectively. These tables focus on the primary validity metric, MrgC, and the prediction set efficiency metrics: AvgC, OneC, and EmpC. Cells with a red background indicate MrgC values below the required 1−α threshold; cells with an violet background flag AvgC values exceeding half the total class space, signaling excessively large prediction sets. Visualizations of AvgC results across nonconformity scores and models for each dataset are provided in Figure 4, Figure 5 and Figure 6. Exact calibrated quantile thresholds for all model–dataset–nonconformity score combinations at α∈{0.01,0.05,0.10} are provided in Appendix F, Table A2, Table A3, Table A4 and Table A5.

#### 3.3.1. Validity

Validity demands that the MrgC probability is at least 1−α, meaning that the empirical marginal coverage on the test set should probabilistically meet or exceed this target. Observing Table 5, Table 6 and Table 7, we analyze the adherence of each nonconformity score to this principle.

Across the four evaluated object detection models and three datasets with varying class counts (including the background class), most nonconformity scores demonstrated strong empirical effectiveness, with MrgC consistently close to or exceeding the target 1−α. For instance, APS frequently showed coverage of 0.99 or higher at α=0.01 and generally met or exceeded the 1−α target at α=0.05 (e.g., Faster R-CNN on MS-COCO-20, Table 5). While the theoretical guarantee is exact in the limit with exchangeability [46], observed minor shortfalls slightly below the target for some methods (e.g., Hinge, Margin, and Brier on Faster R-CNN for P1.5 at α=0.10, Table 6) are typically small fluctuations attributable to finite-sample effects from the calibration set, rather than a fundamental failure of the guarantee. Formally, split conformal prediction admits an exact finite-sample characterization: for exchangeable data, the training-conditional coverage $P(Y_{n+1}\in C\left(X_{n+1}\right)|D_{n})$ stochastically dominates a Beta((1−α)(n+1),α(n+1)) distribution, which yields the concentration bound $P\left(\mathrm{coverage}\leq 1-\alpha -\Delta \right)\leq e^{-2n\Delta^{2}}$ [46]. This Beta law provides an exact reference band for the observed shortfalls. To quantify this, we evaluated each undercoverage case against the Beta reference by computing $P_{\mathrm{Beta}}$, the probability of observing coverage at or below the reported value. For YOLO11, the four undercoverage combinations (Hinge, Margin, and Brier on P1.5v2 at α=0.10, and Hinge on Chula at α=0.05) have $P_{\mathrm{Beta}}\approx 0.02$–0.06 (Table 8); that is, the observed coverage lies at roughly the 2nd–6th percentile of the predicted finite-sample band, consistent with expected finite-sample fluctuation rather than a systematic failure of the guarantee. These tables provide mere empirical snapshots; complete verification of validity ideally involves assessing coverage distributions over multiple i.i.d. trials or different data splits [26]. In contrast, the Naive method, which lacks a calibration step, showed less consistent coverage. It noticeably dips below the target on other datasets (e.g., Faster R-CNN and YOLO11 on Chula at α=0.05, Table 7).

Standard ICP guarantees coverage on average over the full joint distribution of detections and classes, without conditioning on the identity of any specific detection or class. Class-conditional coverage is a strictly stronger property. It requires the guarantee to hold individually for every semantic category, which standard ICP does not ensure. As shown in Appendix E (Figure A6, Figure A7 and Figure A8), these two properties are empirically distinct; per-class coverage rates vary across classes and can fall below the marginal target, illustrating that satisfying the marginal guarantee is not sufficient to ensure class-level coverage. Achieving class-conditional guarantees requires a fundamentally different approach, such as Mondrian conformal prediction [27], which stratifies the calibration set by class label and estimates a separate quantile threshold $\hat{q}$ per class.

#### 3.3.2. Adaptivity and Prediction Set Efficiency

Although not directly captured by the marginal coverage (MrgC) metric, adaptivity is a crucial property for the practical utility and interpretability of conformal prediction sets. It refers to the ability of prediction set sizes to adjust dynamically to the difficulty of individual instances, yielding smaller, more informative sets for easy-to-classify examples and larger sets for more ambiguous or uncertain cases. This instance-wise adaptivity thus reflects the confidence of the model in a transparent manner. Our analysis of the AvgC, OneC, and empty set rates provides insight into the efficiency of the methods and their potential for practical adaptability.

##### AvgC

AvgC is an essential metric for evaluating the efficiency and practical utility of prediction sets, as smaller sets are more informative. The values obtained for this metric indicate substantial variation that is directly correlated with the number of classes in the dataset and the choice of nonconformity score. On the 21-class MS-COCO-20 dataset, the larger output space made it inherently challenging to form small sets while maintaining high coverage. As shown in Table 5, the non-conformal prediction method (Naive) produced large AvgC values, particularly for RetinaNet and RT-DETRv2. For instance, RT-DETERv2 yielded AvgC values of 20.91, 18.01, and 15.10 for α=0.01, 0.05, and 0.1, respectively. In contrast, the conformal variant of Naive (APS) substantially reduced these sizes by factors of approximately 6.70, 9.10, and 9.34. This illustrates how conformal prediction can tune the overly conservative behavior of Naive. Among the other conformal prediction methods, Margin and Brier score also resulted in notably large sets at lower α levels, e.g., for Faster R-CNN at α=0.01, AvgC was approximately 18, reflecting their difficulty in distinguishing the true class from 21 alternatives (including the background). On the other hand, APS and Hinge consistently yielded much smaller AvgC values on MS-COCO-20, demonstrating improved efficiency across models and α levels. Top-K also produced compact sets; however, its set size is fixed by construction and, therefore, is not adaptable to the difficulty of individual instances.

On the five-class P1.5v2 dataset, where the reduced class space simplified the task, all methods produced notably small AvgC, often clustering between 1.00 and 2.00 (Table 6). Notably, regardless of its non-adaptivity, Top-K frequently achieved the minimum possible AvgC of 1.00 on P1.5 at higher α levels, indicating exact singleton predictions. The 12-class Chula dataset presented an intermediate challenge, yielding AvgC values typically between those of MS-COCO-20 and P1.5 (Table 7). Across this range of difficulty, APS and Hinge consistently demonstrated a strong balance between validity and efficiency, yielding considerably smaller AvgC compared to the Naive, Margin, and Brier score methods at lower α levels. Their efficiency in controlling the prediction set size while maintaining coverage across varying class-space complexities is a critical advantage for usability. Although Top-K also produced compact sets, it is essential to note that it is non-adaptive: its set size is fixed by construction. It therefore does not adjust to the difficulty of individual test instances. Figure 4, Figure 5 and Figure 6 visualize the AvgC across nonconformity scores and models for each dataset.

##### OneC

**Figure A3:**
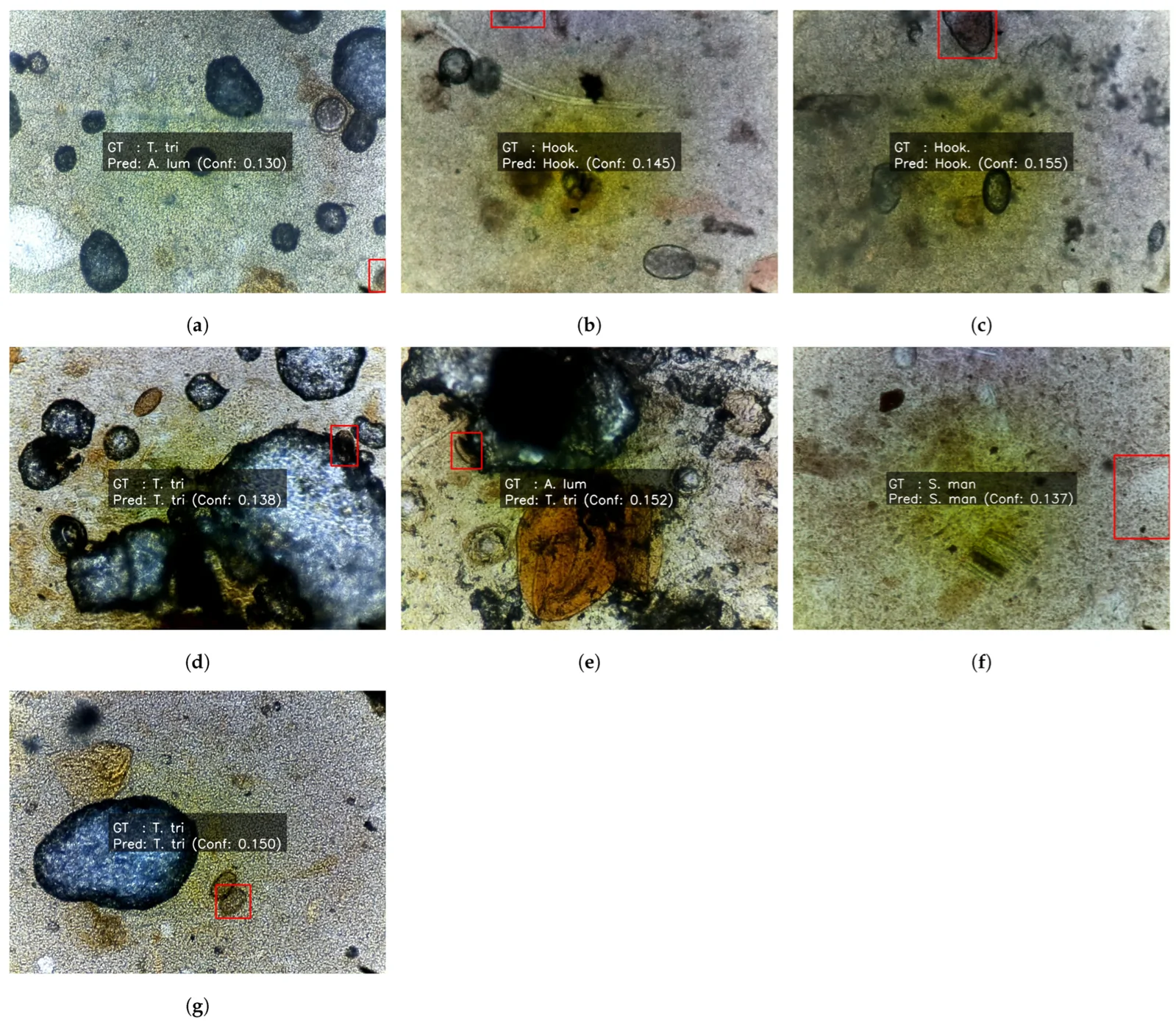
Visual breakdown of the seven missed foreground singletons assigned to the background class. Red bounding boxes indicate predicted regions. Center translucent overlays summarize ground-truth (GT) and predicted (Pred) classes along with initial confidence scores (0.130≤Conf≤0.155). Failure modes are categorized by edge truncation (**a**–**c**), debris occlusion (**d**,**e**), and morphological ambiguity (**f**,**g**).

**Figure A6:**
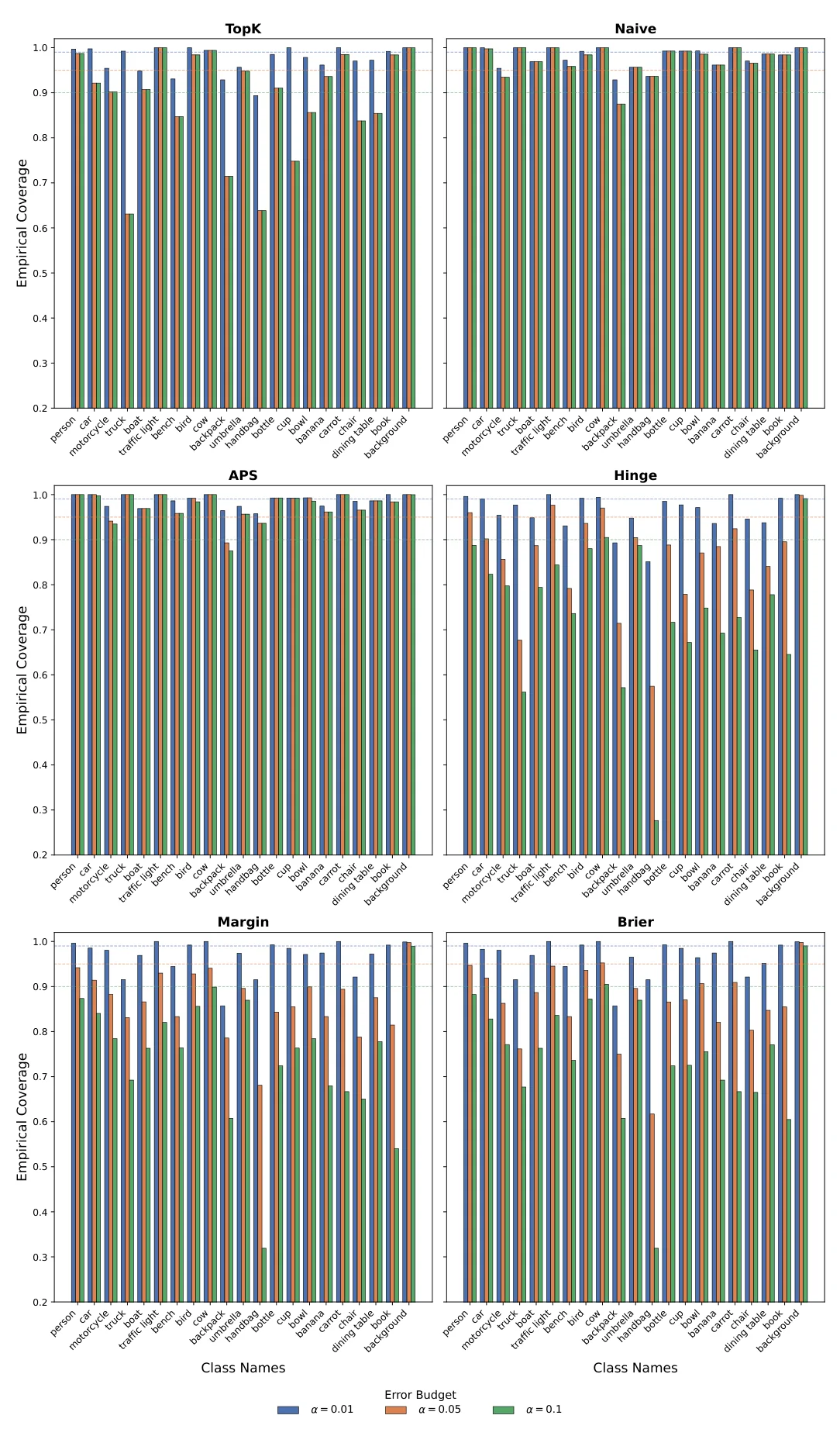
Class-conditional empirical coverage across different nonconformity scoring methods using YOLO11 on the MS-COCO-20 dataset. Each panel compares six scoring functions (TopK, Naive, APS, Hinge, Margin, and Brier) evaluated at target significance levels α∈{0.01,0.05,0.10}. Dashed horizontal lines indicate the theoretical coverage guarantees (1−α).

**Figure A7:**
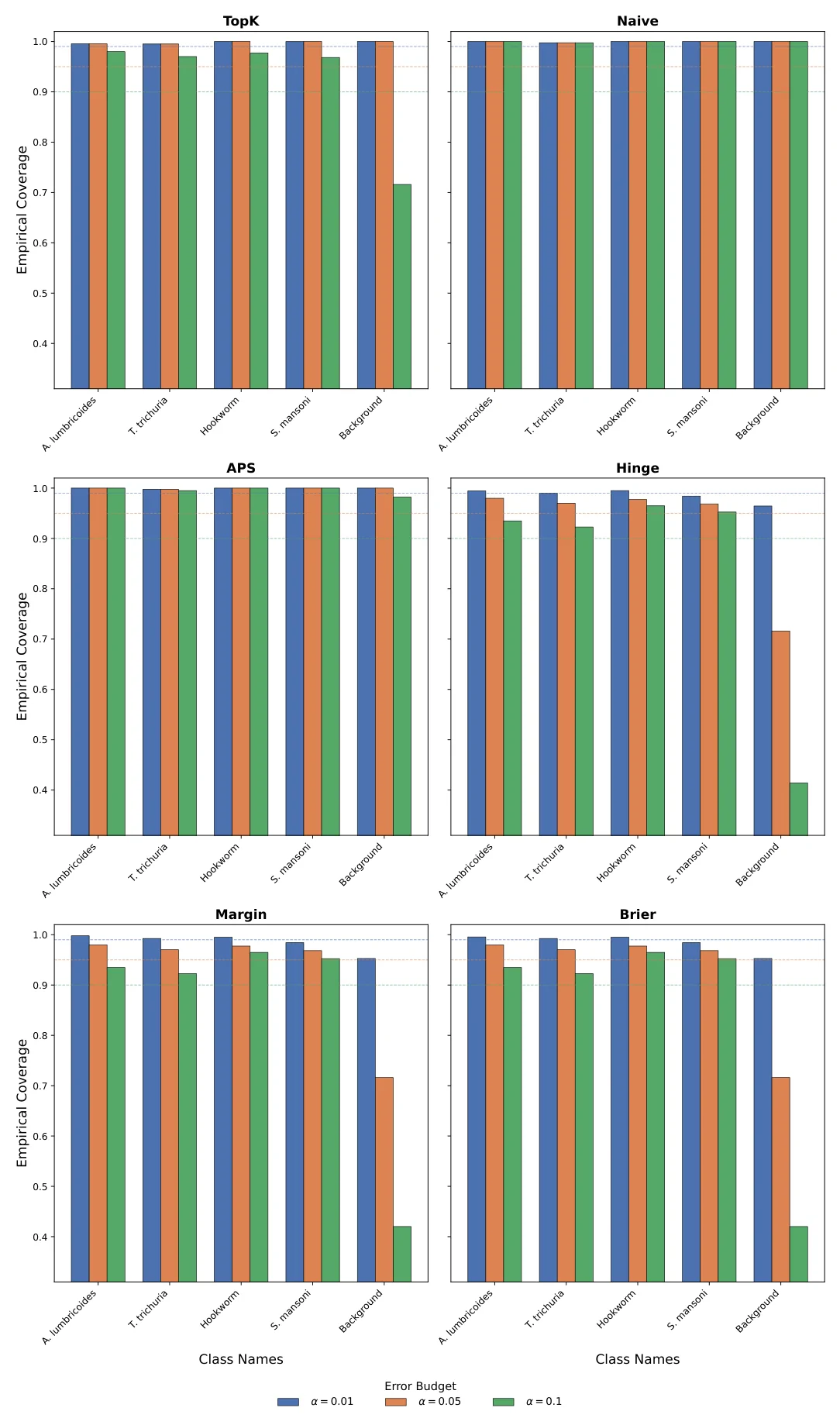
Class-conditional empirical coverage across different nonconformity scoring methods using YOLO11 on the P1.5v2 dataset. Each panel compares six scoring functions (TopK, Naive, APS, Hinge, Margin, and Brier) evaluated at target significance levels α∈{0.01,0.05,0.10}. Dashed horizontal lines indicate the theoretical coverage guarantees (1−α).

**Figure A8:**
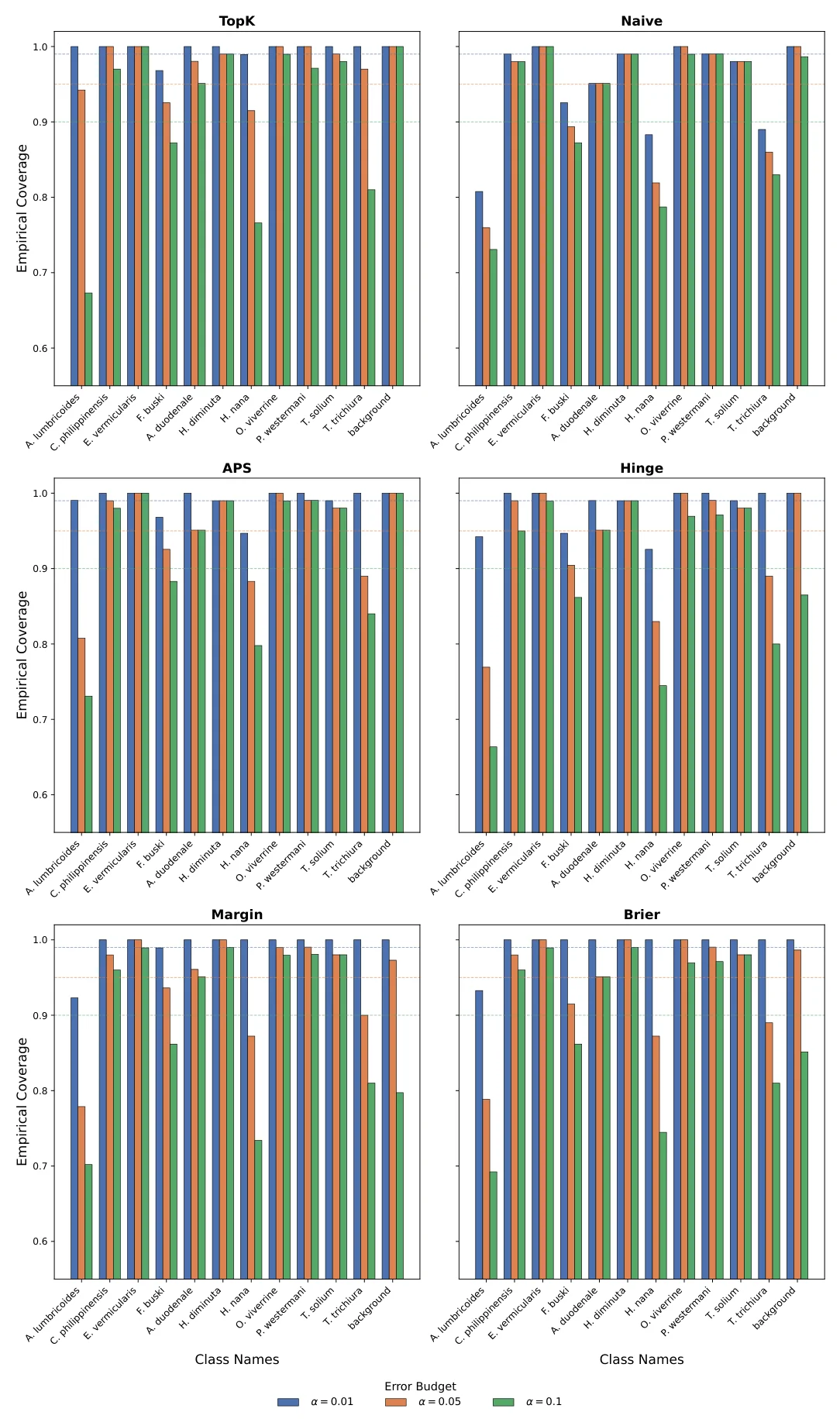
Class-conditional empirical coverage across different nonconformity scoring methods using YOLO11 on the Chula dataset. Each panel compares six scoring functions (TopK, Naive, APS, Hinge, Margin, and Brier) evaluated at target significance levels α∈{0.01,0.05,0.10}. Dashed horizontal lines indicate the theoretical coverage guarantees (1−α).

OneC measures the proportion of predictions that contain exactly a single class label, indicating high precision and confidence in the decision provided that the required marginal coverage is met. In our setting, a single-element prediction set (|C(X)| = 1) is categorized as an informative indicator of classification precision specifically when that element corresponds to a foreground target class; singletons containing exclusively the background class reflect confident candidate rejection rather than positive target identification. Empirically, for YOLO11 on P1.5v2 at α=0.01 under the Margin nonconformity score, 86.54% (45/52) of all background singletons corresponded directly to non-object background proposals (correctly rejecting false-positive candidates). The remaining 13.46% (7/52) stemmed from base detector misclassifications of foreground objects as background. Qualitative inspection of these seven missed singletons (Appendix C, Figure A3) reveals that all produced initial candidate confidence scores within a narrow sub-threshold band (0.130≤Conf≤0.155), primarily driven by physical edge truncation (3/7), severe debris occlusion (2/7), or high morphological ambiguity (2/7). The number of classes markedly impacted the achievable singleton rate: it was higher on the 5-class P1.5v2 dataset than on the 12-class Chula or 21-class MS-COCO-20, as distinguishing the true class from fewer distractors is inherently easier. On P1.5, many conformal prediction methods, especially Hinge, Margin, and Brier score at higher α, achieved exceptionally high, often perfect, OneC (e.g., Table 6, YOLO11, α=0.05: Hinge/Margin/Brier 1.00), indicating near-perfect precision for predictions provided at these confidence levels. On MS-COCO-20 and Chula, OneC values were comparatively lower, reflecting the increased difficulty. Within these larger class spaces, Hinge, Margin, and Brier generally showed greater precision (higher OneC) than the Naive method and APS at higher α levels (e.g., Table 7, RT-DETRv2, α=0.10: Hinge 0.97, Margin 0.98, Brier 0.98 vs Naive 0.00, APS 0.64), demonstrating their ability to generate concise sets when the underlying model scores allow. A notable example is RT-DETRv2 on MS-COCO-20, where Margin achieved an exceptional 1.00 OneC at α=0.10 (Table 5), illustrating that when a strong base model provides well-separated scores, these functions can yield maximum precision even in a large class space. The non-conformal prediction method (Naive) consistently exhibited low OneC, a direct consequence of its general tendency to produce large, inefficient sets on our benchmark datasets.

##### EmpC

EmpC indicates the percentage of instances where the prediction set is empty, representing a failure to provide any prediction. A key strength observed across the evaluation is the exceptionally low frequency of empty sets. For Top-K, Naive, and APS, the EmpC was 0.00% across all datasets, models, and significance levels. By design, these three methods are guaranteed to produce non-empty prediction sets. Hinge, Margin, and Brier score also predominantly achieved 0.00% EmpC but showed minor non-zero values in specific contexts. This was most notable on the five-class P1.5v2 dataset, where EmpC ranged from 0.01 to 0.11 across different models and α values (e.g., Table 6). Minimal empty sets (0.01–0.02%) were also observed for Brier score on RT-DETRv2 for MS-COCO-20 at α=0.10, and for Margin and Brier score on RT-DETRv2 for Chula at α=0.10 (Table 5 and Table 6). This minor occurrence of empty sets for Hinge, Margin, and Brier score, particularly on the simpler P1.5v2 dataset, stems directly from high calibration confidence setting a strict nonconformity threshold. On P1.5v2, due to the relative simplicity and highly curated nature of the dataset, base models such as YOLO11 output near-perfect top-1 confidence across calibration samples. This causes bounded score functions like Brier score to yield near-zero nonconformity values, compressing the calibrated coverage threshold to a very tight cutoff (e.g., a nonconformity threshold of $\hat{q}=0.0258$). During testing, if a candidate proposal exhibits even a modest drop in confidence (such as top-1 confidence dropping to 88%), its nonconformity score exceeds the threshold so that no class meets the inclusion criterion, causing all classes to be rejected and resulting in an empty prediction set. Conversely, on complex datasets like MS-COCO-20 (21 classes), lower base confidence yields larger calibration nonconformity scores, maintaining a wider threshold that absorbs test-time confidence drops. More generally, empty prediction sets signal predictions that fail to meet the calibrated certainty threshold, either due to high classification ambiguity (a uniform confidence spread across classes) or slight confidence drops under strict quantile bounds. Crucially, empty sets are more likely to occur at higher significance levels (larger α), where higher error tolerance yields a smaller quantile threshold $\hat{q}$ that is harder for low-confidence proposals to satisfy. Empty sets arising on background-labeled detections represent principled rejections of non-object regions; notably, their occurrence is exclusively confined to score functions that threshold true-class nonconformity scores directly (Hinge, Margin, Brier), whereas Top-K, Naive, and APS produce non-empty sets by construction.

#### 3.3.3. Subjective Analysis

**Figure 7:**
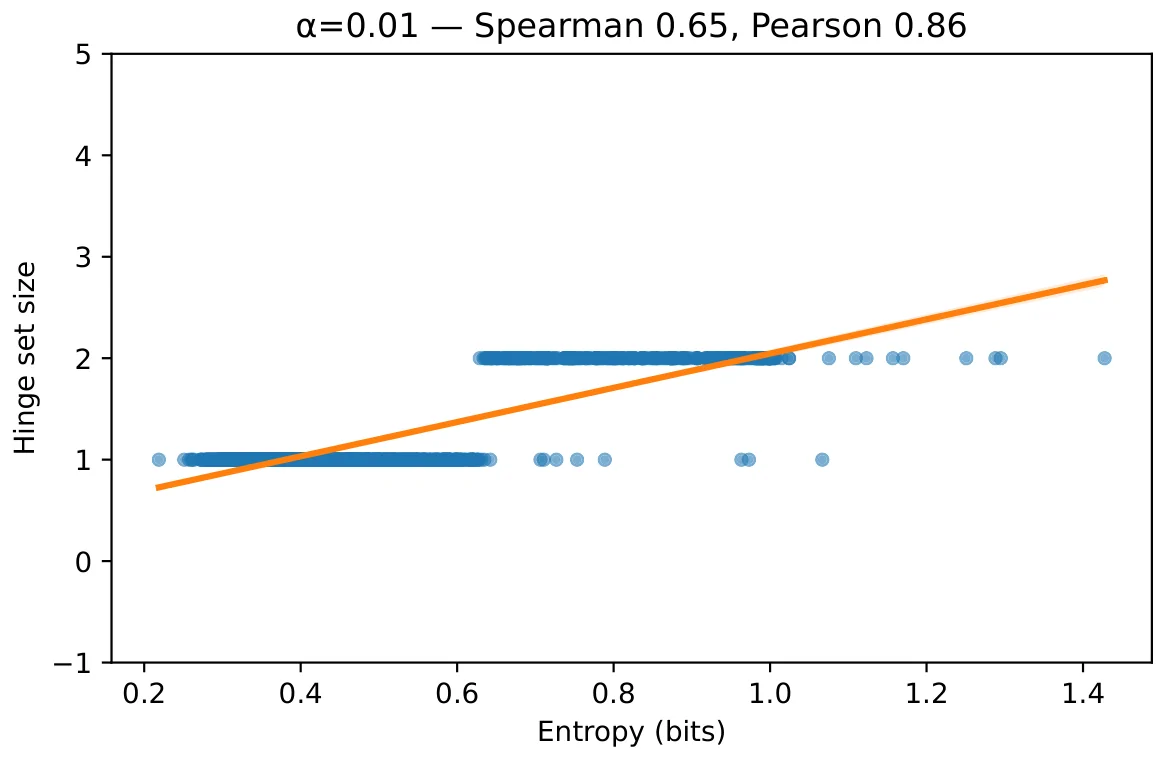
Instance-level adaptivity for YOLO11 with the Hinge nonconformity score at α=0.01 on the P1.5v2 dataset. Each point is a test detection, the horizontal axis is the Shannon entropy of the normalized per-class score vector, and the vertical axis is the conformal prediction set size. The orange line indicates the linear fit highlighting the trend of increasing set size with higher uncertainty. Set size increases monotonically with entropy (Spearman ρ=0.65, Pearson r=0.86; p≪0.001), confirming that larger sets are assigned to more uncertain detections.

**Figure 8:**
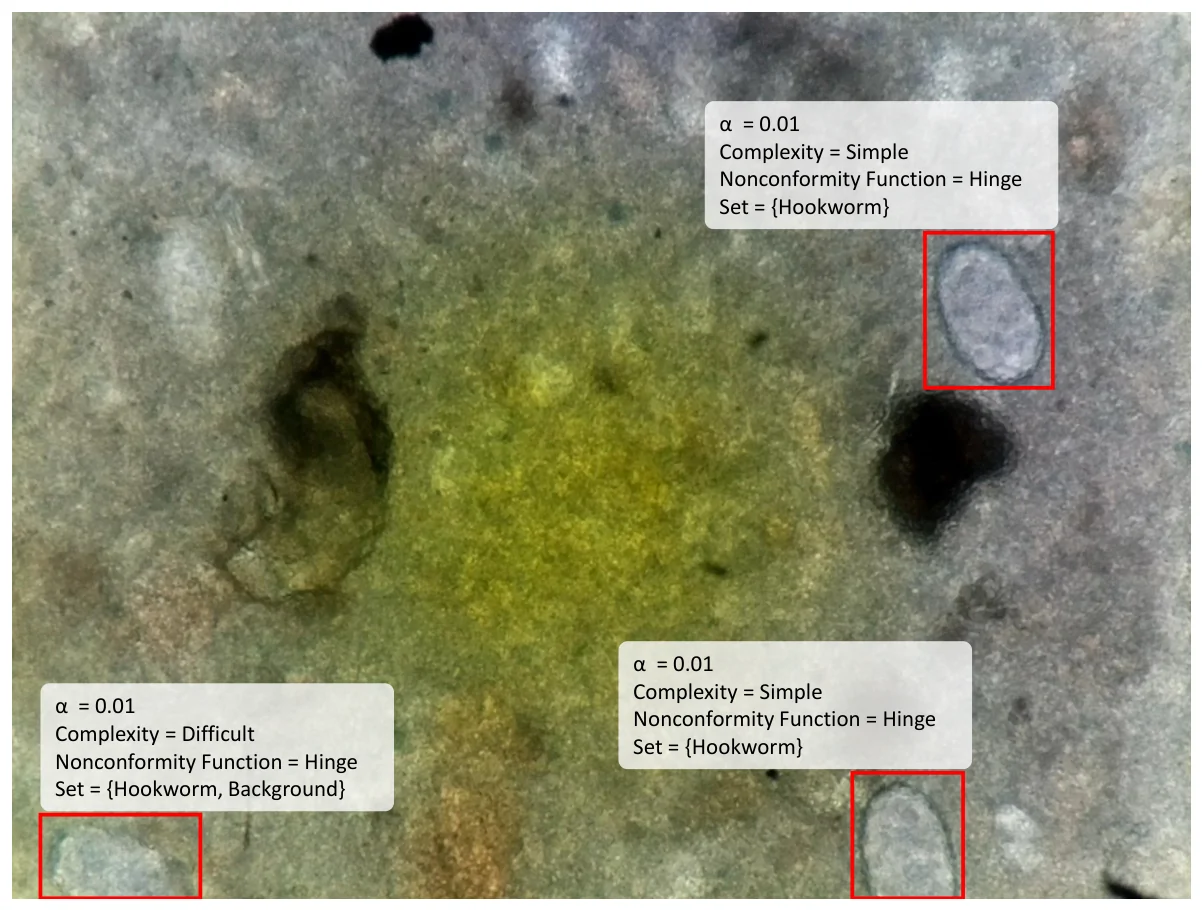
Qualitative examples for the P1.5 dataset at (α=0.01). The instances on the right are straightforward hookworm eggs, resulting in singleton prediction sets. In contrast, the instance in the lower-left is a slightly difficult hookworm egg and requires a larger prediction set to maintain coverage.

While aggregate metrics like MrgC and AvgC are essential, they do not fully characterize the effectiveness of the nonconformity scores. A truly adaptive conformal procedure should yield prediction set sizes that reflect instance-level difficulty: smaller for straightforward samples, larger for complex ones. Without this property, efficiency metrics alone can be misleading. Therefore, both quantitative and qualitative assessments are required to verify that prediction set sizes genuinely correlate with instance complexity, thereby ensuring meaningful adaptivity. To assess this quantitatively, we relate the prediction set size of each detection to its per-detection uncertainty, measured using Shannon entropy as a representative proxy for distribution dispersion computed over the normalized per-class score vector, for YOLO11 using the Hinge nonconformity score at a significance level of α=0.01 on the P1.5v2 dataset—a combination chosen for its reasonable empirical performance and the practical relevance of the parasite detection use case. Set size increases monotonically with entropy (Spearman ρ=0.65, Pearson r=0.86; p≪0.001), confirming that larger sets are assigned to more uncertain detections rather than being distributed arbitrarily, as shown in Figure 7. Complementing this, Figure 8 visually illustrates the prediction set behavior for the same configuration, providing a representative real-world setting in which to demonstrate meaningful adaptivity. Visualizations for the remaining two datasets (Ms-COCO-20 and Chula) are added in Appendix D.

#### 3.3.4. Comparison with MC-Dropout

While MC-Dropout offers a Bayesian approximation to uncertainty quantification by leveraging stochastic forward passes to assess model consistency, its practical application is often hampered by substantial computational overhead. Specifically, MC-Dropout necessitates multiple inferences for a single image (e.g., T=10) with active dropout layers, leading to a significant increase in computational cost. The uncertainty generated by the MC-Dropout pipeline is quantified by aggregating the empirical distribution across the *T* stochastic forward passes. For a given input image, the final classification score is computed as the empirical mean of the predicted probability vectors. The epistemic uncertainty (or model hesitation) is subsequently derived by calculating either the empirical variance or the predictive entropy across these *T* discrete samples. High variance among the outputs indicates that the active dropout masks are disrupting unstable internal representations, signaling low model confidence. Conversely, a tightly clustered output distribution indicates a robust, high-confidence prediction. However, the computational overhead imposed by the *T* passes makes the method impractical for real-time applications and deployment on resource-constrained edge devices, where latency and power consumption are critical.

In stark contrast, ICP presents a highly efficient and rigorously guaranteed alternative for uncertainty estimation. ICP operates on the output of a single, deterministic forward pass, introducing negligible overhead while providing robust finite-sample marginal coverage guarantees. This ensures that the true class label is contained within the prediction set at a user-specified significance level α. While a direct comparison between MC-Dropout and ICP evaluates two epistemologically distinct paradigms of uncertainty, characterizing their respective operational efficiencies provides a critical metric for practical utility. Furthermore, ICP is entirely model-agnostic, requiring no architectural modifications, specialized loss functions, or retraining. This inherent flexibility and minimal computational footprint position ICP as a feasible solution for edge-device applications. MC-Dropout was chosen as a benchmark because it also provides post hoc uncertainty without model retraining. However, its overhead scales linearly with the number of forward passes, unlike the constant single-pass overhead of ICP. Empirical evidence supporting these efficiency claims is shown in Table 9 for the SOTA YOLO11 model only. Among the five nonconformity scores evaluated, APS incurred a modest latency overhead of approximately <2.0%. In contrast, MC-Dropout introduced an overhead exceeding 800%, making the computational cost of all nonconformity scores negligible by comparison.

#### 3.3.5. Implications of the Research

In summary, selecting a nonconformity function requires balancing set adaptivity, runtime latency, and empty-set risks. APS is recommended as the primary default for maximum adaptivity to instance ambiguity without yielding empty sets. For latency-critical applications, Hinge is optimal, delivering the lowest runtime overhead while preserving adaptivity. Conversely, Top-K is not recommended due to its fixed, non-adaptive nature and higher processing latency relative to Hinge. Finally, while Margin and Brier score achieve tight set-size efficiency, practitioners should exercise caution, as they can suffer from empty prediction sets on highly saturated datasets.

Building on these operational trade-offs, the successful application of conformal prediction to the semantic classification component of object detection—which allows seamless post hoc integration across standard, fine-tuned models without architectural modifications, although switching detector architectures requires brief recalibration due to model-specific effectiveness dynamics—carries practical and scientific implications. This capability, which provides statistically guaranteed class-label coverage through prediction sets, fundamentally transforms the trustworthiness and utility of object detection systems in real-world scenarios as follows:

- *Enhanced Reliability in Automated Systems:* By providing prediction sets with statistically guaranteed coverage, conformal prediction enables automated systems to not only produce predictions but also quantify their uncertainty in a principled manner. Instances with higher uncertainty, as indicated by larger prediction sets, can be automatically flagged for further examination. This allows for the strategic allocation of human expert intervention to review, validate, or correct only the most ambiguous or high-risk instances. Such targeted oversight enhances overall system reliability while maintaining scalability, particularly in sensitive applications such as medical diagnosis, where misclassification (such as parasite identification and counting) can have critical consequences. Moreover, the ability to signal uncertainty in predictions enhances transparency and trust in automated decision-making pipelines, paving the way for more responsible, human-in-the-loop AI deployment.
- *Streamlined Data Annotation and Curation:* The enhanced trustworthiness and interpretability of object detection outputs, particularly through adaptive methods that differentiate between easy and hard examples based on prediction set size, offer potential for streamlining labor-intensive tasks such as data annotation and dataset curation. By clearly identifying instances where the model is highly confident (small sets) versus uncertain (large sets), resources can be efficiently prioritized for validation and cleaning efforts, focusing human attention where it is most needed.
- *Broadened Applicability in Critical Domains:* The demonstration that this framework can be applied to standard, fine-tuned object detection models without requiring complex architectural modifications or extensive retraining is a key advantage. This facilitates immediate integration into existing pipelines across various critical domains, providing a solid foundation for downstream tasks that require reliable uncertainty estimates. Specifically, prediction sets act directly as calibrated candidate lists, allowing single-element sets to pass automatically while routing multi-label sets for expert review. Such adaptability is crucial for deployment in applications where regulatory or ethical considerations mandate predictable uncertainty behavior, such as biomedical imaging.
- *Foundation for Future Research and Development:* This work establishes a solid empirical foundation for further research into uncertainty quantification for object detection, particularly concerning the semantic class-label component. It opens avenues for exploring more refined nonconformity scores specifically tailored to the nuances of object detection outputs related to semantic differentiation, including the development of non-heuristic methods for background class confidence score estimation, investigating instance-conditional coverage properties for semantic labels in more detail, and developing strategies to improve the efficiency of prediction sets on highly complex datasets while maintaining coverage guarantees.

## 4. Conclusions

This work addressed the challenge of quantifying semantic uncertainty in object detection by introducing an ICP framework that provides probabilistically guaranteed class-label coverage for standard detectors. We demonstrated that widely used models—including Faster R-CNN, RetinaNet, YOLO11, and RT-DETRv2—can be conformalized without architectural changes or retraining by exposing per-class confidence scores and, when necessary, generating a synthetic background-class score. To support systematic evaluation, we also introduced MS-COCO-20, a curated 20-class benchmark for assessing semantic uncertainty quantification methods.

Comprehensive experiments across MS-COCO-20 and two parasitological datasets (P1.5v2 and Chula) confirmed the validity of the framework and revealed clear efficiency differences across nonconformity scores. On the highly curated P1.5v2 dataset, conformal prediction produced highly efficient singleton sets, while the more challenging Chula and MS-COCO-20 datasets highlighted the ability of the framework to adapt to varying task complexities. Across all datasets, all conformal methods consistently achieved or closely approached the target marginal coverage (1−α), with minor deviations attributable to finite-sample effects.

Efficiency analysis showed that adaptive nonconformity scores, particularly Hinge and APS, offered the best balance between coverage and set size. While Top-K provided minimal but non-adaptive sets, adaptive scores produced smaller sets when model confidence allowed and expanded them appropriately under stricter coverage requirements. Methods such as Hinge, Margin, and Brier score achieved high efficiency but were more sensitive to dataset complexity and base-model choice. Importantly, all valid conformal methods exhibited a negligible empty set rate, underscoring their practical reliability.

By equipping standard object detectors with prediction sets that carry statistical coverage guarantees, this work enables more trustworthy deployment in safety-critical settings. Larger prediction sets naturally flag uncertain classifications for targeted human review, supporting applications such as parasite egg identification and improving data annotation workflows by prioritizing ambiguous instances.

Future research should explore principled approaches for estimating background confidence scores, methods that relax the i.i.d. assumption, and techniques that emphasize marginal rather than strict instance-conditional coverage, including class-conditional coverage guarantees for rare and imbalanced class populations via Mondrian conformal prediction [47]. Because the present experiments primarily establish ICP’s applicability in object detection, a more principled stability validation using repeated resampling across splits is deferred to future work. Investigating robustness under distribution shift (including the open-set scenarios where test classes fall outside the calibration set) and integrating conformal prediction with Explainable AI, such as designing nonconformity scores that reveal why a prediction is uncertain, represents a promising direction for advancing both reliability and interpretability in object detection.

## Figures and Tables

**Table 1:** Object instance distribution within the MS-COCO-20 dataset, presented per class and split (train, validation, calibration, test) for use in inductive conformal prediction experiments.

Object	Train	Val.	Cal.	Test
Person	23,282	5717	2604	2853
Car	6036	1583	652	636
Motorcycle	2127	450	145	226
Truck	1885	493	221	186
Boat	2251	556	216	196
Traffic Light	2252	480	231	288
Bench	1680	410	200	202
Bird	2302	644	212	213
Cow	1808	507	161	219
Backpack	1640	409	194	177
Umbrella	2458	591	221	189
Handbag	2079	484	241	232
Bottle	2575	727	320	243
Cup	2162	484	256	238
Bowl	2029	387	242	201
Banana	1841	599	203	176
Carrot	1559	538	199	172
Chair	3153	630	483	530
Dining Table	1695	399	188	262
Book	1898	531	274	342

**Table 2:** Object instance distribution within the AI4NTD KK2.0 P1.5 STH & SCHm v2 (P1.5v2) dataset, presented per class (egg type) and split (train, validation, calibration, test), for use in inductive conformal prediction experiments.

Parasite (Egg Type)	Train	Val.	Cal.	Test
*A. lumbricoides*	5097	1305	1766	899
*T. trichuria*	2342	586	904	406
Hookworm	2080	521	760	412
*S. mansoni*	381	96	143	65

**Table 3:** Object instance distribution within the Chula-ParasiteEgg-11 (Chula) dataset, presented per class (egg type) and split (train, validation, calibration, test), for use in inductive conformal prediction experiments.

Parasite (Egg Type)	Train	Val.	Cal.	Test
*A. lumbricoides*	907	102	101	106
*C. philippinensis*	900	100	102	101
*E. vermicularis*	900	100	100	100
*F. buski*	900	100	100	99
Hookworm	910	100	102	102
*H. diminuta*	100	100	100	100
*H. nana*	901	100	100	102
*O. viverrine*	900	100	100	100
*Paragonimus* spp.	900	100	103	106
*Taenia* spp.	911	100	103	101
*T. trichiura*	900	100	100	100

**Table 4:** Object detection models used in this study, with summaries of their detection paradigms, architectures, and classification activations.

Name	Detection Paradigm	Architecture	Classification Activation
Faster R-CNN [38]	Two-stage	DCNN	Softmax
RetinaNet [39]	One-stage	DCNN	Sigmoid
YOLO11 [40]	One-stage	DCNN	Sigmoid
RT-DETRv2 [41]	One-stage	Transformer	Sigmoid

**Table 5:** Effectiveness of the nonconformity scores on the MS-COCO-20 dataset, evaluated at three significance levels: α=0.01, α=0.05, and α=0.1. Red background indicates MrgC values below 1−α; violet background indicates AvgC values exceeding half the total class space.

		Faster R-CNN					
Metric	α	Top-K	Naive	APS	Hinge	Margin	Brier
MrgC	0.01	1.00	0.99	1.00	0.99	0.99	0.99
	0.05	0.98	0.96	0.98	0.95	0.95	0.95
	0.10	0.98	0.94	0.96	0.90	0.90	0.90
AvgC	0.01	3.00	2.27	2.83	2.18	18.00	17.99
	0.05	2.00	1.91	2.04	1.81	10.23	8.32
	0.10	2.00	1.81	1.88	1.50	4.46	1.53
OneC	0.01	0.00	0.09	0.04	0.09	0.12	0.12
	0.05	0.00	0.18	0.13	0.22	0.25	0.24
	0.10	0.00	0.23	0.19	0.51	0.53	0.52
EmpC	0.01	0.00	0.00	0.00	0.00	0.00	0.00
	0.05	0.00	0.00	0.00	0.00	0.00	0.00
	0.10	0.00	0.00	0.00	0.00	0.00	0.00
		**RetinaNet**					
MrgC	0.01	1.00	1.00	1.00	0.99	0.99	0.99
	0.05	0.98	1.00	0.98	0.95	0.95	0.95
	0.10	0.98	1.00	0.97	0.90	0.89	0.90
AvgC	0.01	3.00	17.37	4.15	2.09	11.19	8.16
	0.05	2.00	10.20	2.22	1.32	1.94	1.31
	0.10	2.00	5.77	1.70	1.07	1.07	1.07
OneC	0.01	0.00	0.00	0.01	0.05	0.18	0.16
	0.05	0.00	0.00	0.18	0.69	0.72	0.71
	0.10	0.00	0.01	0.42	0.92	0.94	0.93
EmpC	0.01	0.00	0.00	0.00	0.00	0.00	0.00
	0.05	0.00	0.00	0.00	0.00	0.00	0.00
	0.10	0.00	0.00	0.00	0.00	0.00	0.00
		**YOLO11**					
MrgC	0.01	0.99	0.99	0.99	0.99	0.99	0.99
	0.05	0.97	0.98	0.98	0.95	0.95	0.95
	0.10	0.97	0.97	0.97	0.90	0.90	0.90
AvgC	0.01	4.00	2.27	2.94	2.48	13.09	12.08
	0.05	2.00	2.06	2.11	1.86	8.21	2.62
	0.10	2.00	2.00	2.03	1.56	3.76	1.56
OneC	0.01	0.00	0.00	0.00	0.00	0.02	0.02
	0.05	0.00	0.00	0.00	0.16	0.26	0.21
	0.10	0.00	0.02	0.01	0.44	0.49	0.46
EmpC	0.01	0.00	0.00	0.00	0.00	0.00	0.00
	0.05	0.00	0.00	0.00	0.00	0.00	0.00
	0.10	0.00	0.00	0.00	0.00	0.00	0.00
		**RT-DETRv2**					
MrgC	0.01	1.00	1.00	1.00	0.99	0.99	0.99
	0.05	0.99	1.00	0.98	0.95	0.95	0.95
	0.10	0.99	1.00	0.97	0.90	0.90	0.90
AvgC	0.01	3.00	20.91	3.12	2.07	10.08	6.68
	0.05	2.00	18.01	1.98	1.26	1.72	1.20
	0.10	2.00	15.10	1.61	1.01	1.00	1.00
OneC	0.01	0.00	0.00	0.00	0.05	0.23	0.22
	0.05	0.00	0.00	0.38	0.74	0.85	0.81
	0.10	0.00	0.00	0.60	0.96	1.00	0.98
EmpC	0.01	0.00	0.00	0.00	0.00	0.00	0.00
	0.05	0.00	0.00	0.00	0.00	0.00	0.00
	0.10	0.00	0.00	0.00	0.01	0.00	0.01

**Table 6:** Effectiveness of the nonconformity scores on the P1.5v2 dataset, evaluated at three significance levels: α=0.01, α=0.05, and α=0.1. Red background indicates MrgC values below 1−α; violet background indicates AvgC values exceeding half the total class space.

		Faster R-CNN					
Metric	α	Top-K	Naive	APS	Hinge	Margin	Brier
MrgC	0.01	1.00	0.99	0.99	0.99	0.99	0.99
	0.05	0.96	0.99	0.99	0.94	0.94	0.94
	0.10	0.96	0.98	0.99	0.89	0.89	0.89
AvgC	0.01	2.00	1.09	1.13	1.09	1.30	1.30
	0.05	1.00	1.06	1.07	0.96	0.96	0.96
	0.10	1.00	1.05	1.05	0.90	0.89	0.89
OneC	0.01	0.00	0.91	0.89	0.91	0.91	0.91
	0.05	1.00	0.94	0.94	0.96	0.96	0.96
	0.10	1.00	0.95	0.95	0.90	0.89	0.89
EmpC	0.01	0.00	0.00	0.00	0.00	0.00	0.00
	0.05	0.00	0.00	0.00	0.04	0.04	0.04
	0.10	0.00	0.00	0.00	0.10	0.11	0.11
		**RetinaNet**					
MrgC	0.01	0.99	1.00	1.00	0.99	0.99	0.99
	0.05	0.95	0.99	0.99	0.95	0.95	0.95
	0.10	0.95	0.99	0.99	0.89	0.89	0.89
AvgC	0.01	2.00	2.31	2.05	1.16	1.50	1.51
	0.05	1.00	1.35	1.27	0.99	1.00	1.00
	0.10	1.00	1.18	1.17	0.91	0.90	0.91
OneC	0.01	0.00	0.36	0.45	0.85	0.85	0.84
	0.05	1.00	0.74	0.79	0.99	1.00	1.00
	0.10	1.00	0.84	0.84	0.91	0.90	0.91
EmpC	0.01	0.00	0.00	0.00	0.00	0.00	0.00
	0.05	0.00	0.00	0.00	0.01	0.00	0.00
	0.10	0.00	0.00	0.00	0.09	0.10	0.09
		**YOLO11**					
MrgC	0.01	1.00	1.00	1.00	0.99	0.99	0.99
	0.05	1.00	1.00	1.00	0.95	0.95	0.95
	0.10	0.95	1.00	0.99	0.89	0.89	0.89
AvgC	0.01	2.00	2.01	2.00	1.17	1.32	1.16
	0.05	2.00	1.99	1.32	1.00	1.00	1.00
	0.10	1.00	1.35	1.18	0.91	0.91	0.91
OneC	0.01	0.00	0.00	0.01	0.83	0.84	0.84
	0.05	0.00	0.02	0.69	1.00	1.00	1.00
	0.10	1.00	0.65	0.82	0.91	0.91	0.91
EmpC	0.01	0.00	0.00	0.00	0.00	0.00	0.00
	0.05	0.00	0.00	0.00	0.00	0.00	0.00
	0.10	0.00	0.00	0.00	0.09	0.09	0.09
		**RT-DETRv2**					
MrgC	0.01	1.00	1.00	1.00	0.99	0.99	0.99
	0.05	1.00	1.00	1.00	0.95	0.95	0.95
	0.10	0.95	1.00	0.99	0.90	0.90	0.90
AvgC	0.01	2.00	4.44	1.84	1.23	1.53	1.44
	0.05	2.00	2.38	1.40	1.01	1.01	1.01
	0.10	1.00	1.66	1.27	0.92	0.92	0.92
OneC	0.01	0.00	0.00	0.30	0.80	0.78	0.78
	0.05	0.00	0.00	0.64	0.99	0.99	0.99
	0.10	1.00	0.44	0.75	0.92	0.92	0.92
EmpC	0.01	0.00	0.00	0.00	0.00	0.00	0.00
	0.05	0.00	0.00	0.00	0.00	0.00	0.00
	0.10	0.00	0.00	0.00	0.08	0.08	0.08

**Table 7:** Effectiveness of the nonconformity functions on the Chula-ParasiteEgg11 dataset, evaluated at three significance levels: α=0.01, α=0.05, and α=0.1. Red background indicates MrgC values below 1−α; violet background indicates AvgC values exceeding half the total class space.

		Faster R-CNN					
Metric	α	Top-K	Naive	APS	Hinge	Margin	Brier
MrgC	0.01	0.99	0.98	0.99	0.99	0.99	0.99
	0.05	0.96	0.94	0.97	0.94	0.96	0.95
	0.10	0.96	0.93	0.94	0.89	0.90	0.90
AvgC	0.01	4.00	1.80	2.98	1.99	4.53	4.54
	0.05	2.00	1.30	1.58	1.29	2.72	2.68
	0.10	2.00	1.19	1.30	1.06	1.11	1.06
OneC	0.01	0.00	0.68	0.46	0.61	0.64	0.64
	0.05	0.00	0.80	0.73	0.80	0.81	0.81
	0.10	0.00	0.85	0.80	0.95	0.95	0.94
EmpC	0.01	0.00	0.00	0.00	0.00	0.00	0.00
	0.05	0.00	0.00	0.00	0.00	0.00	0.00
	0.10	0.00	0.00	0.00	0.00	0.00	0.00
		**RetinaNet**					
MrgC	0.01	0.99	1.00	0.99	0.98	0.99	0.99
	0.05	0.95	0.99	0.97	0.93	0.93	0.93
	0.10	0.92	0.98	0.96	0.88	0.87	0.87
AvgC	0.01	5.00	7.86	5.29	3.40	6.35	6.36
	0.05	3.00	4.40	2.73	1.43	1.78	1.46
	0.10	2.00	2.90	2.05	1.05	1.05	1.04
OneC	0.01	0.00	0.03	0.16	0.31	0.46	0.45
	0.05	0.00	0.23	0.39	0.69	0.84	0.79
	0.10	0.00	0.37	0.51	0.94	0.96	0.96
EmpC	0.01	0.00	0.00	0.00	0.00	0.00	0.00
	0.05	0.00	0.00	0.00	0.00	0.00	0.00
	0.10	0.00	0.00	0.00	0.00	0.00	0.00
		**YOLO11**					
MrgC	0.01	1.00	0.99	0.99	0.99	0.99	0.99
	0.05	0.98	0.93	0.95	0.94	0.95	0.95
	0.10	0.91	0.92	0.93	0.90	0.90	0.90
AvgC	0.01	5.00	2.20	3.20	2.82	7.65	7.87
	0.05	3.00	2.00	2.20	2.13	2.61	2.43
	0.10	2.00	1.48	1.57	1.13	1.17	1.14
OneC	0.01	0.00	0.00	0.00	0.00	0.02	0.01
	0.05	0.00	0.09	0.00	0.00	0.72	0.67
	0.10	0.00	0.56	0.49	0.88	0.90	0.88
EmpC	0.01	0.00	0.00	0.00	0.00	0.00	0.00
	0.05	0.00	0.00	0.00	0.00	0.00	0.00
	0.10	0.00	0.00	0.00	0.00	0.00	0.00
		**RT-DETRv2**					
MrgC	0.01	1.00	1.00	0.99	0.99	0.99	0.99
	0.05	0.97	1.00	0.97	0.95	0.95	0.94
	0.10	0.90	1.00	0.96	0.90	0.90	0.90
AvgC	0.01	5.00	11.31	4.20	3.20	5.84	5.93
	0.05	3.00	8.20	1.88	1.29	1.50	1.21
	0.10	1.00	5.40	1.57	0.98	0.98	0.98
OneC	0.01	0.00	0.00	0.00	0.14	0.42	0.37
	0.05	0.00	0.00	0.48	0.80	0.91	0.90
	0.10	0.00	0.00	0.64	0.97	0.98	0.98
EmpC	0.01	0.00	0.00	0.00	0.00	0.00	0.00
	0.05	0.00	0.00	0.00	0.00	0.00	0.00
	0.10	0.00	0.00	0.00	0.02	0.02	0.02

**Table 8:** Statistical validation of YOLO11 coverage deviations. Δ=(1−α)−MrgC; $P_{\mathrm{Beta}}$ is the Beta((1−α)(n+1),α(n+1)) CDF at the observed coverage (Theorem 4.1, [46]); the last column is the $e^{-2n\Delta^{2}}$ bound.

Dataset	Score	α	1−α	MrgC	Δ	$n_{\mathrm{cal}}$	$P_{\mathrm{Beta}}$	$e^{-2n\Delta^{2}}$
P1.5v2	Hinge	0.10	0.90	0.89	0.01	3857	0.02	0.46
P1.5v2	Margin	0.10	0.90	0.89	0.01	3857	0.02	0.46
P1.5v2	Brier	0.10	0.90	0.89	0.01	3857	0.02	0.46
Chula	Hinge	0.05	0.95	0.94	0.01	1179	0.06	0.79

**Table 9:** Empirical runtime overhead comparison between MC-Dropout (T=10) and inductive conformal prediction (ICP) for YOLO11 across datasets. Base detector runtime per image is P1.5v2 = 15.42±0.10 ms, MS-COCO-20 = 17.16±0.32 ms, Chula = 14.22±0.04 ms (isolated pure inference and NMS post-processing).

Dataset	Method	Conform/MC (ms)	ms/Image	Overhead (%)
P1.5v2	MC-Dropout (T=10)	106.58±0.58	119.36±0.60	833.96
	ICP:			
	Top-K	72.82±0.87	0.097	0.63
	Naive	168.87±1.21	0.225	1.46
	APS	225.20±0.63	0.299	1.94
	Hinge	22.69±0.34	0.030	0.19
	Margin	58.71±0.29	0.078	0.51
	Brier	69.26±0.23	0.092	0.60
MS-COCO-20	MC-Dropout (T=10)	124.22±3.12	138.84±3.32	849.66
	ICP:			
	Top-K	92.48±0.88	0.092	0.53
	Naive	213.75±1.62	0.212	1.24
	APS	284.14±2.50	0.282	1.64
	Hinge	28.42±0.31	0.028	0.16
	Margin	102.25±1.26	0.101	0.59
	Brier	111.95±0.99	0.111	0.65
Chula	MC-Dropout (T=10)	105.33±0.47	118.14±0.55	822.25
	ICP:			
	Top-K	100.83±0.81	0.091	0.64
	Naive	166.48±1.62	0.151	1.06
	APS	241.91±1.51	0.219	1.54
	Hinge	32.35±0.25	0.029	0.21
	Margin	86.00±0.32	0.078	0.55
	Brier	98.96±0.47	0.089	0.63

**Figure A1:**
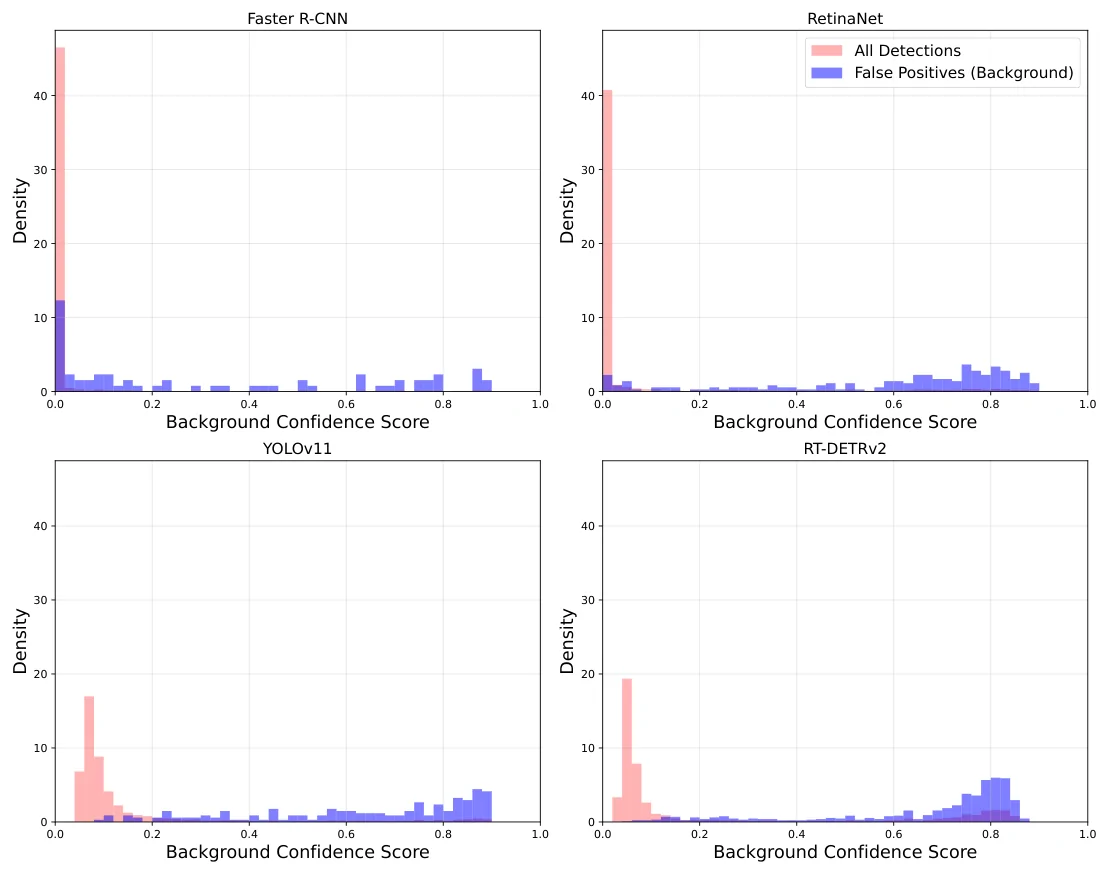
Distribution of background confidence scores on the P1.5 test set for Faster R-CNN, RetinaNet, YOLO11, and RT-DETRv2. Overlaid histograms show “All Detections” and “False Positives (Background)”.

**Figure A2:**
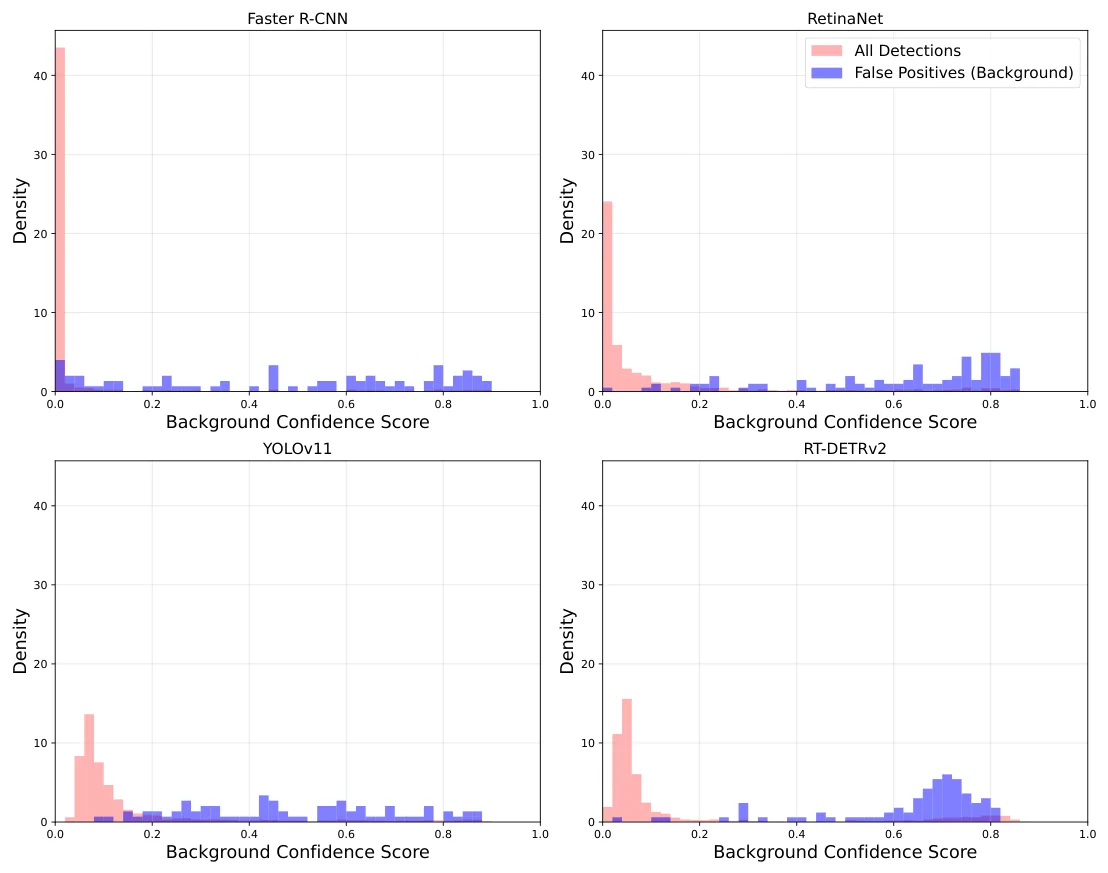
Distribution of background confidence scores on the Chula test set for Faster R-CNN, RetinaNet, YOLO11, and RT-DETRv2. Overlaid histograms show “All Detections” and “False Positives (Background)”.

**Figure A4:**
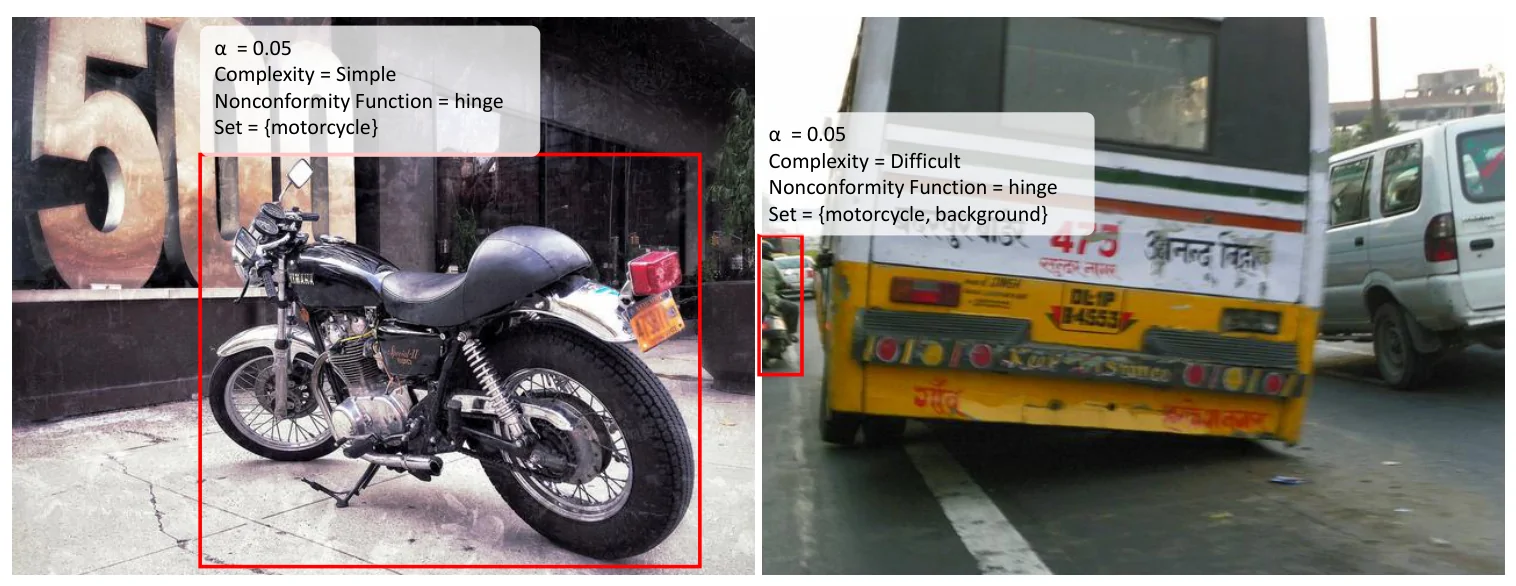
Qualitative assessment of prediction set adaptivity. This figure shows the behavior of our conformal framework with a YOLOv11 model and a Hinge nonconformity score on the MS-COCO-20 dataset (α=0.05). The **left** column presents a straightforward motorcycle instance, which results in a singleton prediction set. The **right** column presents an ambiguous motorcycle instance, resulting in a two-element prediction set to maintain target coverage.

**Figure A5:**
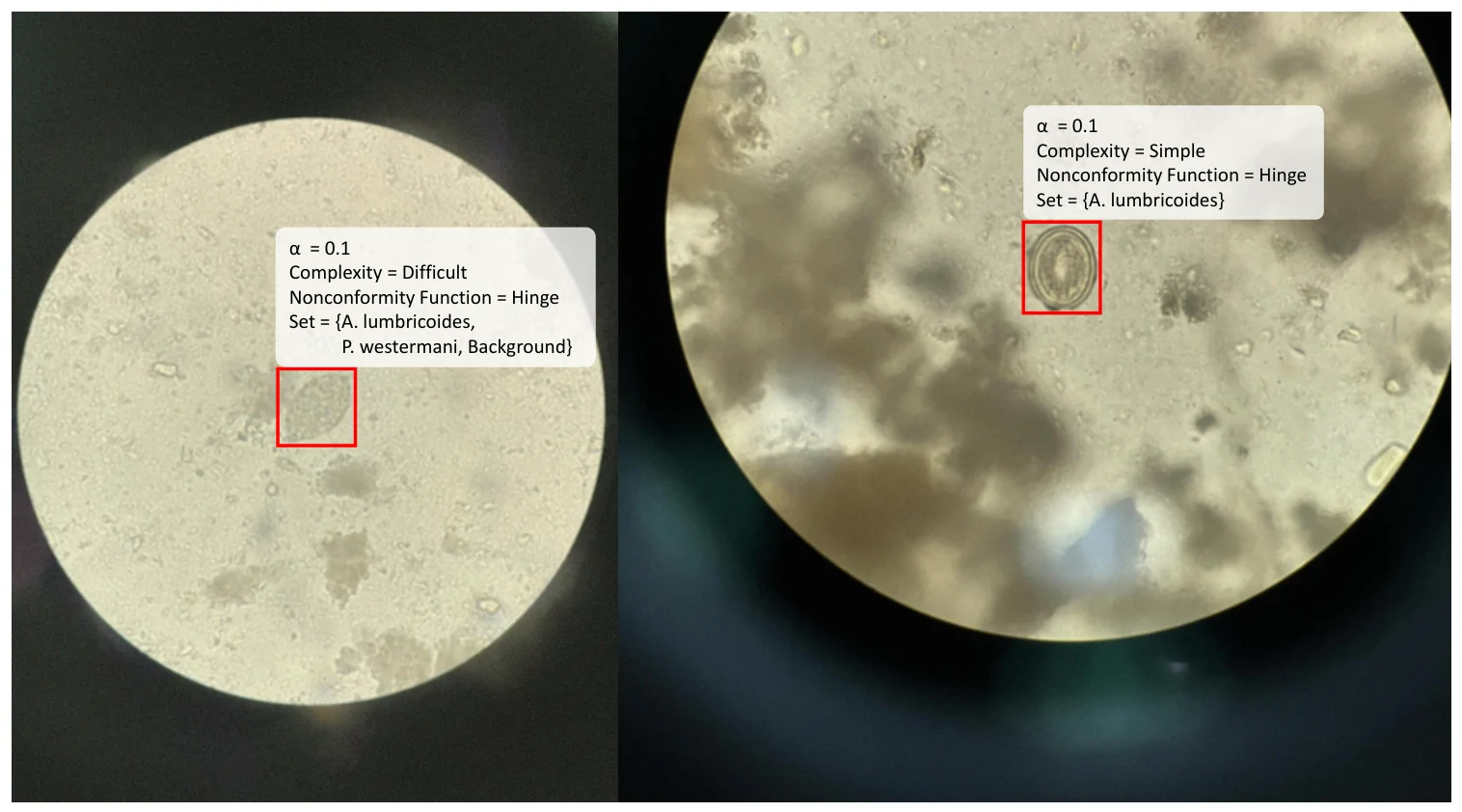
Qualitative examples for the Chula dataset at (α=0.1). The instance in the **right** column is a straightforward *A. lumbricoides* egg, resulting in a singleton prediction set. In contrast, the instance in the **left** column is a slightly difficult *A. lumbricoides* egg and requires a larger prediction set to maintain coverage.

## Data Availability

The dataset supporting this study is publicly available in Dataset for Conformal Object Detection at the following DOI: https://doi.org/10.34740/kaggle/ds/7567820, accessed on 31 July 2026.

## Abbreviations

The following abbreviations are used in this manuscript:

AI	Artificial intelligence
AP	Average precision
APS	Adaptive Prediction Sets
AvgC	Average set size
BOF	Special Research Fund
Cal.	Calibration
Chula	Chula-ParasiteEgg-11
DCNNs	Deep Convolutional Neural Networks
EDL	Evidential deep learning
EmpC	Empty set rate
ICP	Inductive conformal prediction
IoU	Intersection over Union
MC	Monte Carlo
MrgC	Marginal coverage
MS-COCO-20	Curated 20-class subset derived from MS-COCO
NCFs	Nonconformity functions
OOD	Out-of-distribution
OneC	Average singleton sets
P1.5v2	AI4NTD KK2.0 P1.5 STH & SCHm v2
Val.	Validation
i.i.d.	Independently and identically distributed

## Appendix A. Baseline Model Performance

**Table A1:** Performance metrics (mAP@IoU_0.5_, mAP@IoU_0.5:0.95_, and mAR@IoU_0.5:0.95_) for the base object detection models across the MS-COCO-20, P1.5v2, and Chula datasets for the validation, calibration, and test splits.

		mAP@IoU_0.5_			mAP@IoU_0.5:0.95_			mAR@IoU_0.5:0.95_		
Dataset	Model	Val.	Cal.	Test	Val.	Cal.	Test	Val.	Cal.	Test
MS-COCO-20	Faster R-CNN	0.576	0.472	0.483	0.351	0.282	0.289	0.472	0.401	0.406
	RetinaNet	0.576	0.447	0.463	0.359	0.266	0.279	0.485	0.391	0.402
	YOLO11	0.524	0.377	0.398	0.354	0.228	0.240	0.477	0.378	0.401
	RT-DETRv2	0.449	0.526	0.469	0.284	0.338	0.295	0.448	0.503	0.477
P1.5v2	Faster R-CNN	0.964	0.972	0.972	0.838	0.843	0.835	0.875	0.881	0.870
	RetinaNet	0.981	0.980	0.982	0.854	0.851	0.836	0.884	0.883	0.870
	YOLO11	0.990	0.994	0.988	0.863	0.902	0.854	0.977	0.990	0.955
	RT-DETRv2	0.990	0.994	0.988	0.860	0.901	0.843	0.964	0.987	0.970
Chula	Faster R-CNN	0.982	0.886	0.871	0.906	0.813	0.790	0.933	0.840	0.822
	RetinaNet	0.986	0.885	0.865	0.918	0.821	0.796	0.939	0.845	0.822
	YOLO11	0.953	0.994	0.948	0.870	0.935	0.864	0.913	0.993	0.899
	RT-DETRv2	0.960	0.994	0.954	0.891	0.941	0.879	0.903	0.994	0.891

## Appendix F. Calibrated Quantile Thresholds

**Table A2:** Calibrated quantile thresholds $\hat{q}$ for Faster R-CNN across datasets and nonconformity scores.

Dataset	$n_{\mathrm{cal}}$	Score	α=0.01	α=0.05	α=0.10
P1.5v2	3630	Hinge	0.9905	0.1407	0.0039
P1.5v2	3630	Margin	0.9783	−0.7283	−0.9930
P1.5v2	3630	Brier	0.3914	0.0079	0.0000
P1.5v2	3630	Naive	0.9900	0.9500	0.9000
P1.5v2	3630	APS	0.9974	0.9676	0.9204
P1.5v2	3630	TopK	1	0	0
MS-COCO-20	17,901	Hinge	0.9929	0.9191	0.8241
MS-COCO-20	17,901	Margin	0.9671	0.7892	0.6143
MS-COCO-20	17,901	Brier	0.0922	0.0766	0.0626
MS-COCO-20	17,901	Naive	0.9900	0.9500	0.9000
MS-COCO-20	17,901	APS	0.9969	0.9764	0.9397
MS-COCO-20	17,901	TopK	2	1	1
Chula	1153	Hinge	0.9973	0.9724	0.7847
Chula	1153	Margin	0.9893	0.9034	0.4952
Chula	1153	Brier	0.1649	0.1510	0.0986
Chula	1153	Naive	0.9900	0.9500	0.9000
Chula	1153	APS	0.9978	0.9828	0.9504
Chula	1153	TopK	3	1	1

**Table A3:** Calibrated quantile thresholds $\hat{q}$ for RetinaNet across datasets and nonconformity scores.

Dataset	$n_{\mathrm{cal}}$	Score	α=0.01	α=0.05	α=0.10
P1.5v2	3898	Hinge	0.9321	0.5139	0.2401
P1.5v2	3898	Margin	0.8165	−0.0324	−0.5873
P1.5v2	3898	Brier	0.3311	0.0949	0.0190
P1.5v2	3898	Naive	0.9900	0.9500	0.9000
P1.5v2	3898	APS	0.9858	0.9352	0.8920
P1.5v2	3898	TopK	1	0	0
MS-COCO-20	28,945	Hinge	0.9332	0.8175	0.6844
MS-COCO-20	28,945	Margin	0.6567	0.3953	0.1253
MS-COCO-20	28,945	Brier	0.0664	0.0487	0.0323
MS-COCO-20	28,945	Naive	0.9900	0.9500	0.9000
MS-COCO-20	28,945	APS	0.8658	0.7637	0.6939
MS-COCO-20	28,945	TopK	2	1	1
Chula	1197	Hinge	0.9792	0.9023	0.7319
Chula	1197	Margin	0.8302	0.4138	0.1204
Chula	1197	Brier	0.1399	0.0946	0.0594
Chula	1197	Naive	0.9900	0.9500	0.9000
Chula	1197	APS	0.9661	0.8920	0.8366
Chula	1197	TopK	4	2	1

**Table A4:** Calibrated quantile thresholds $\hat{q}$ for YOLO11 across datasets and nonconformity scores.

Dataset	$n_{\mathrm{cal}}$	Score	α=0.01	α=0.05	α=0.10
P1.5v2	3857	Hinge	0.8425	0.5019	0.2550
P1.5v2	3857	Margin	0.6588	0.0012	−0.4927
P1.5v2	3857	Brier	0.2752	0.1002	0.0258
P1.5v2	3857	Naive	0.9900	0.9500	0.9000
P1.5v2	3857	APS	0.9525	0.8939	0.8464
P1.5v2	3857	TopK	1	1	0
MS-COCO-20	8602	Hinge	0.9977	0.8792	0.8082
MS-COCO-20	8602	Margin	0.8056	0.7053	0.5742
MS-COCO-20	8602	Brier	0.0791	0.0710	0.0607
MS-COCO-20	8602	Naive	0.9900	0.9500	0.9000
MS-COCO-20	8602	APS	0.9980	0.9701	0.9236
MS-COCO-20	8602	TopK	3	1	1
Chula	1179	Hinge	0.9998	0.9830	0.7672
Chula	1179	Margin	0.9242	0.7374	0.4217
Chula	1179	Brier	0.1548	0.1300	0.0884
Chula	1179	Naive	0.9900	0.9500	0.9000
Chula	1179	APS	0.9997	0.9902	0.9131
Chula	1179	TopK	4	2	1

**Table A5:** Calibrated quantile thresholds $\hat{q}$ for RT-DETRv2 across datasets and nonconformity scores.

Dataset	$n_{\mathrm{cal}}$	Score	α=0.01	α=0.05	α=0.10
P1.5v2	4752	Hinge	0.8746	0.6206	0.3253
P1.5v2	4752	Margin	0.6833	0.0936	−0.4547
P1.5v2	4752	Brier	0.2845	0.1261	0.0325
P1.5v2	4752	Naive	0.9900	0.9500	0.9000
P1.5v2	4752	APS	0.9109	0.8614	0.8135
P1.5v2	4752	TopK	1	1	0
MS-COCO-20	60,334	Hinge	0.9394	0.8840	0.7686
MS-COCO-20	60,334	Margin	0.4722	0.2280	0.0048
MS-COCO-20	60,334	Brier	0.0556	0.0445	0.0318
MS-COCO-20	60,334	Naive	0.9900	0.9500	0.9000
MS-COCO-20	60,334	APS	0.6043	0.5265	0.4843
MS-COCO-20	60,334	TopK	2	1	1
Chula	1181	Hinge	0.9744	0.8997	0.6484
Chula	1181	Margin	0.7561	0.3773	−0.0502
Chula	1181	Brier	0.1304	0.0880	0.0479
Chula	1181	Naive	0.9900	0.9500	0.9000
Chula	1181	APS	0.8724	0.7869	0.7458
Chula	1181	TopK	4	2	0

Notes. In Table A2, Table A3, Table A4 and Table A5 (1) Naive thresholds are 1−α by construction. (2) TopK is zero-based: 0 = top-1, 1 = top-2, 2 = top-3, etc.
